# Conditions for improved accuracy of noninvasive preimplantation genetic testing for aneuploidy: Focusing on the zona pellucida and early blastocysts

**DOI:** 10.1002/rmb2.12604

**Published:** 2024-09-10

**Authors:** Hiroki Takeuchi, Midori Morishita, Midori Uemura, Tadashi Maezawa, Takashi Shibahara, Erina Takayama, Mikiko Nishioka, Eiji Kondo, Hiroyuki Minoura, Tomoaki Ikeda

**Affiliations:** ^1^ Department of Obstetrics and Gynecology, Graduate School of Medicine Mie University Tsu Japan; ^2^ Center of Advanced Reproductive Medicine Mie University Hospital Tsu Japan; ^3^ IVF Shiroko Clinic Suzuka Japan; ^4^ Minoura Ladies Clinic Suzuka Japan; ^5^ Department of Obstetrics and Gynecology Mie University Hospital Tsu Japan

**Keywords:** blastocyst, embryo, niPGT‐A, PGT‐A, zona pellucida

## Abstract

**Purpose:**

Recently, noninvasive preimplantation genetic testing for aneuploidy (niPGT‐A) using cell‐free deoxyribonucleic acid has been developed; however, there are few reports on this and the results are inconsistent. This study was conducted to optimize the cultural environment.

**Methods:**

We used 35 blastocysts that had been discarded after in‐vitro fertilization. The concordance rate of karyotype analysis results between whole embryos (WEs), spent culture mediums (SCMs), and trophectoderms after 8, 16, and 24 h of culture was examined. Next, zona pellucida (ZP)‐free blastocysts and then early blastocysts were cultured for 24 h each.

**Results:**

Regarding the optimal culture times, the concordance rate between WEs and SCMs was 20%, 60%, and 100% at 8, 16, and 24 h, respectively. Significant differences were found between 8 and 24 h. The concordance rate with ZP cultures was 40.0%, and no significant differences were found. The concordance rate of early blastocysts thawed and cultured for 24 h was 40.0%, which was significantly lower than that of day 5 blastocysts.

**Conclusions:**

The optimal culture times for niPGT‐A were 24 h, and the concordance rate with free ZP was higher. The concordance rate for early blastocysts was low, suggesting that optimization of the conditions may be necessary.

## Graphical abstract

The following factors were found to be important in improving the accuracy of noninvasive preimplantation genetic testing for aneuploidy (niPGT‐A) in this study: embryos should be cultured for at least 24 h; zona pellucida is required during culture; and the number of contractions during culture. The accuracy of the niPGT‐A results obtained from day 4 early blastocysts cultured for 24 h was also validated.

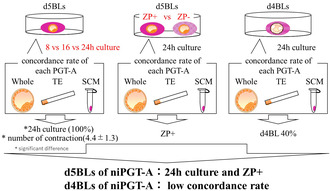

## 1 INTRODUCTION

Chromosomal aberrations in human embryos are very high, ranging from 20% to 80%,^1^ and are strictly correlated with female age.^2^ The aneuploidy rate of embryos increases exponentially with increasing maternal age and is a major cause of pregnancy failure, pregnancy loss, and congenital anomalies.^3^, ^4^ One way to overcome these problems is through the use of preimplantation genetic testing for aneuploidy (PGT‐A), in which a portion of a trophectoderm (TE) cell is biopsied and the chromosomes of that cell are analyzed to determine the aneuploidy of the embryo. PGT‐A has been reported to improve the pregnancy rate per embryo transfer and reduce the miscarriage rate in older patients.^5^, ^6^, ^7^ However, there are also reports of no improvement in overall pregnancy rates^8^ pregnancy rates in younger patients^5^ or cumulative birth rates in younger patients^9^; therefore, further accumulation of knowledge is important.

Technically, embryos can have mosaicism, which is a mixture of euploid and aneuploid cells; however, when TE‐biopsied cells are mosaic, it does not always accurately indicate the condition of the embryo.^10^ In fact, there are several reports of healthy babies being born after the transfer of embryos diagnosed with mosaicism,^11^, ^12^, ^13^ and the birth rate has been reported to be over 40%.^14^, ^15^ In addition, TE biopsy in PGT‐A is invasive to the embryo and may affect embryo viability and subsequent development.^16^, ^17^, ^18^ Therefore, a minimally invasive testing method that does not involve embryo biopsy is desirable.

In 1948, cell‐free deoxyribonucleic acid (DNA) (cfDNA) was discovered in human blood,^19^ and in 1997, fetal‐derived cfDNA was discovered in maternal plasma.^20^ Noninvasive prenatal testing has been performed for certain genetic abnormalities in the fetus during pregnancy using cfDNA^21^, ^22^, ^23^ and has become an important method for liquid biopsy. In 2013, embryo‐derived cfDNA was reported to be present in the spent culture mediums (SCMs) of human embryos.^24^ In 2016, noninvasive PGT‐A (niPGT‐A) using next‐generation sequencing (NGS) with SCM without the need for TE biopsy was reported as a minimally invasive embryo evaluation method.^25^ In the same year, niPGT‐A by array comparative genomic hybridization was reported; however, the concordance rate was lower than that of TE biopsy.^26^ Subsequently, several studies have evaluated the efficacy of niPGT‐A. Although niPGT‐A was initially found to be superior to TE biopsy,^27^, ^28^ some studies have recently reported a similar^29^ or up to 93.8% concordance rate with TE biopsy results.^30^ However, further optimization of SCM biopsy techniques is needed for clinical application because of the overwhelming lack of knowledge regarding niPGT‐A.

Among the optimized culture techniques, the culture time of embryos has been intensively studied. It has been reported that changing the medium on day 3 is important to reduce the probability of contamination by maternally derived cells.^25^ However, another study reported that changing the medium on day 4 and incubating for 2–3 days increased the concordance rate.^31^ Other studies have also used culture periods of 24 h or longer from days 3/4 to 5/6, suggesting that a culture period of 1–2 days may provide the most stable results.^29^, ^30^, ^31^, ^32^, ^33^, ^34^, ^35^, ^36^, ^37^, ^38^ However, implantation, clinical pregnancy, and birth rates in blastocyst transfer have been reported to decrease at days 5, 6, and 7, respectively^39^; therefore, the embryo culture times for niPGT‐A should be within 24 h. Currently, there are no reports to our knowledge evaluating the effects of culture times within 24 h, the presence or absence of a clear zone, or the effects of blastocyst dynamics. In addition, when considering blastocysts transferred on day 5 (d5‐BLs), when *in‐vitro* fertilization (IVF) results are the most optimal, niPGT‐A using the SCM cultured from day 4 embryos would be ideal; however, there are few reports that have evaluated only early blastocysts transferred on day 4 (d4‐BL2s).

Therefore, in this study, we aimed to identify the optimal cultural environment by analyzing (1) optimal culture times by evaluating the effect of different culture times on the concordance rate of whole embryo (WE), SCM, and TE chromosome test results; (2) the effect of the presence or absence of the zona pellucida (ZP) at the optimal culture times; (3) the accuracy of the results obtained by culturing embryos from d4‐BL2s for clinical use; and (4) the relationship between the number of blastocyst contractions in each condition (culture times, ZP presence or absence, and d4‐BL2s).

## 2 MATERIALS AND METHODS

### 2.1 Ethical approval

This study was conducted on blastocysts of patients who had completed IVF treatment and which were treated as discarded. Informed consent was obtained from all patients. The study was performed in accordance with the guidelines established by the Clinical Research Ethics Review Committee of Mie University Hospital (H2022‐175), conducted within the guidelines established by the Ethics Committee of the Japan Society of Obstetrics and Gynecology (No. 104), and registered in the Japan Registry of Clinical Trials (jRCT1040220121).

### 2.2 Procedure

**FIGURE 1:**
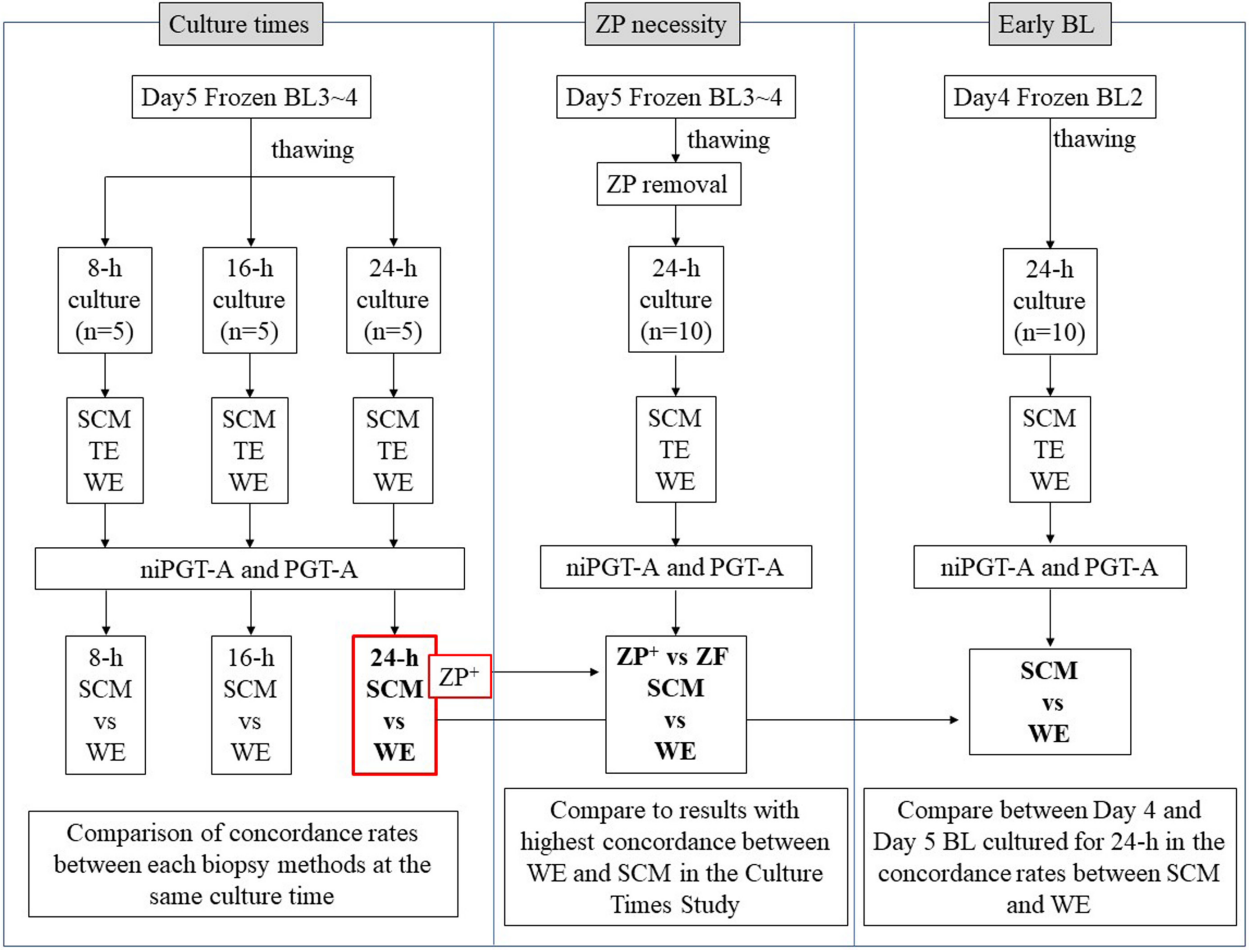
Flow chart of this study. Eight‐hour culture, frozen blastocyst on day 5 thawed and biopsied after 8 h of culture; 16‐h culture, frozen blastocyst on day 5 thawed and biopsied after 16 h of culture; 24‐h culture, frozen blastocyst on day 5 thawed and biopsied after 24 h of culture; SCM, spent culture media; TE, trophectoderm biopsies; WE, whole embryo; ZP+, same as biopsied samples after thawing frozen blastocysts on day 5 and culturing for 24 h; ZF, day 5 blastocysts are thawed and zona pellucida is removed and biopsied after culture for 24 h; BL2, early blastocysts on day 4 are thawed and biopsied after culture for 24 h.

The primary endpoint of this study was the concordance rate of karyotype analysis results between the WE, SCM, and TE of thawed blastocysts (Figure 1). First, the optimal culture times for the SCM were investigated at 8, 16, and 24 h (*n* = 5 for each). Second, to determine the effect of the presence or absence of the ZP, blastocysts with the clear zone removed were cultured at the culture time that showed the highest agreement rate among the SCM culture times described above and were compared to the agreement rate with the clear zone (*n* = 10). Finally, we evaluated the agreement rate when d4‐BL2s were cultured at the culture times that showed the highest agreement rate (*n* = 10).

### 2.3 Embryo background

**FIGURE 2:**
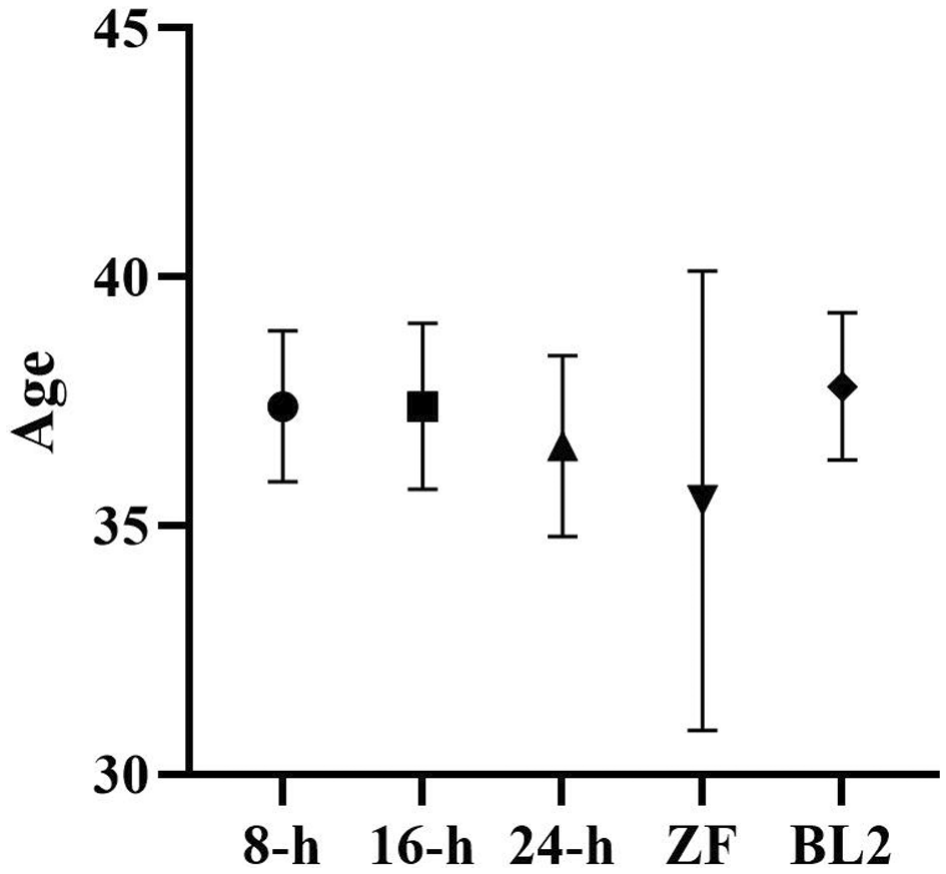
Mean age of eligible patients in each condition. Eight‐hour, frozen blastocyst on day 5 thawed and biopsied after 8 h of culture; 16‐h, frozen blastocyst on day 5 thawed and biopsied after 16 h of culture; 24‐h, frozen blastocyst on day 5 thawed and biopsied after 24 h of culture; ZF, day 5 blastocysts are thawed and zona pellucida is removed and biopsied after culture for 24‐h; BL2, early blastocysts on day 4 are thawed and biopsied after culture for 24 h.

Thirty‐five blastocysts discarded from 24 patients who underwent IVF at Minoura Ladies Clinic and Mie University Hospital by December 2022 were used in this study after obtaining informed consent. Embryos were obtained from patients who had terminated infertility treatment for various reasons, such as having the desired number of children and wishing to discard the embryos. The mean age of the patients was 36.9 ± 2.9 years (Figure 2). We evaluated blastocysts before freezing, at the end of culture, and immediately after TE biopsy using the Gardner Classification.^40^

### 2.4 Embryo thawing and blastocyst culture

Blastocysts were thawed in thawing media (Kitazato) according to the manufacturer's protocol. Assisted hatching was performed while the embryos contracted during thawing, using the LYKOS Clinical IVF laser system and Clinical Laser Software Legacy 5.12 (Hamilton Thorne). Thawed blastocysts were transferred to a multipurpose processing medium (Multipurpose Handling Medium [MHM]; Irvine Scientific) containing 10% v/v dextran serum supplement (DSS; Irvine Scientific) and washed three to four times. The blastocysts were then transferred to a 10 μL drop of HiGROW OVIT PLUS (Fuso Industries) and cultured one at a time in LUX3 FL (Axion Biosystems) at 37°C in 5% CO_2_ and 6% O_2_. Time‐lapse observations were also conducted. Five blastocysts were cultured for 8, 16, and 24 h. After the end of culture, 8 μL of the SCM was sampled into polymerase chain reaction (PCR) tubes according to the manufacturer's (Igenomix Japan) recommended protocol.

Trophectoderm biopsies were performed after the SCM sampling. ZPs were fixed in a drop of 10% DSS‐containing MHM with a holding pipette (CooperSurgical), and five to ten cells were biopsied and collected in PCR tubes, according to the manufacturer's recommended protocol. The WEs were sampled by fixing the ZPs of the blastocysts remaining after TE biopsy with a holding pipette, aspirating and evacuating the cells with a TE biopsy pipette (CooperSurgical), and collecting them in PCR tubes, excluding the ZPs. Blastocysts without ZP (ZP free; ZF) were cultured for 24 h after a short exposure of blastocysts in Acidic Tyrode's solution (Merck) immediately after thawing to remove the ZP, and sampling was performed in the same way. d4‐BL2s were cultured for 24 h immediately after thawing and sampled in the same manner. Each sample was stored at −80°C until analysis, and samples were sent to Igenomix for NGS.

### 2.5 Time‐lapse observation of contraction

Images taken with the LUX3 FL were analyzed with SytoSMART (Axion Biosystems). The time interval was 10 min for time‐lapse observation. Each imaging time was assessed and the number of contractions was counted.

### 2.6 NGS

Whole genome amplification and DNA barcoding were performed using the Ion ReproSeq PGS Kit (Thermo Fisher Scientific), according to the manufacturer's recommendations, using a modified protocol for spent blastocyst medium. Batches of 96 samples were pooled. Nucleic acid extraction was performed using a QIAamp DNA Mini Kit (Qiagen). The extracted DNA was amplified using the ReproSeqTM PGTS kit with Ion 530TM Chips (Thermo Fisher Scientific), and DNA libraries were prepared and sequenced using Ion GeneStudio S5 (Thermo Fisher Scientific). Numerical chromosome aberrations and imbalanced structural aberrations were detected in the obtained sequence data using Ion Reporter Software (ThermoFisher Scientific). In addition, Igenomix's proprietary analysis algorithm was used for PGT‐A^41^ and niPGT‐A.^42^ The samples were quantified and diluted using a Qubit High Sensitivity dsDNA Kit (Life Technologies) and loaded into Ion Chef (ThermoFisher Scientific) for automated template preparation and chip loading. The runs of the 96 samples were performed using 530 chips. Chip sequencing was performed using an S5 sequencer (ThermoFisher Scientific). Aneuploidy and copy number polymorphisms were analyzed using Ion Reporter software (ThermoFisher Scientific). Analysis data after NGS in WEs, SCMs, and TEs were output as described above (Figures S1–S3).

### 2.7 Statistical analysis

In this study, a Fisher's exact test was performed to determine the agreement rate owing to the small sample size. The age and number of contractions were compared using the Dunn's multiple comparison test. All statistical analyses were performed using GraphPad Prism, version 9.4.1 (GraphPad Software); *p* < 0.05 was considered statistically significant.

## 3 RESULTS

### 3.1 Overall findings

In this study, karyotyping was performed on 35 blastocysts via NGS using the WE, SCM, and TE. Patient age, fertilization method, grade, karyotyping results for each sample, and the number of contractions are shown in Table 1. There was no significant difference in patient age between the groups (Figure 2). The blastocysts used in this study were either d5‐BLs or d4‐BL2s before freezing, and most embryos were BL3BB or of higher quality after culture, except for one. Most of the embryos after TE biopsy were BL3BB or of higher quality, except for two.

**TABLE 1:** Comparison of results from 35 samples.

No.	Age	Fertilization method	Grade			Karyotype			Contractions (time)
			Before culture	After culture	After biopsy	WE	SCM	TE	
8–1	35	Rescue ICSI	BL4AB	BL4AB	BL4AB	XY, Euploid	XY, Non‐Info	XY, Euploid	0
8–2	39	ICSI	BL3BB	BL3BB	BL3BB	XX, +20, 21	XX, +4, −10, +20, +21	XX, +20, 21	3
8–3	38	IVF	BL3BB	BL2BB	BL2BB	XY, Euploid	No DNA	XY, Euploid	1
8–4	37	IVF	BL3BB	BL4BA	BL4BA	XY, −7	XY, −7, −17	XY, −7	0
8–5	38	IVF	BL4AA	BL4AA	BL4AA	XY, −16	XY, −16	XY, −16	0
16–1	37	IVF	BL4BB	BL5AA	BL5AA	XY, Euploid	XY, Euploid	XY, Euploid	2
16–2	36	IVF	BL3BB	BL3BB	BL3BC	XX, Euploid	XX, −19	XX, Euploid	2
16–3	36	ICSI	BL4AB	BL4AB	BL4AB	XY, Euploid	XY, Euploid	XY, Euploid	4
16–4	40	IVF	BL4AA	BL4AA	BL4AB	XY, Euploid	XY, Euploid	XY, Complex Mosaic	4
16–5	38	IVF	BL3BB	BL3BB	BL3BB	XY, Euploid	XY, −7, −14	XY, Euploid	3
24–1	35	IVF	BL4AB	BL5AA	BL5AA	XX, +3q	XX, +3q	XX, +3q	3
24–2	35	IVF	BL3BB	BL3BA	BL3BB	XY, −16	XY, −16	XY, −10, 16	6
24–3	39	IVF	BL3BB	BL5BA	BL5BA	XX, Euploid	XX, Euploid	XX, Euploid	6
24–4	36	ICSI	BL4BB	BL4BB	BL4BB	XY, low mosaic −7	XY, low mosaic −7	XY, low mosaic −7	3
24–5	38	IVF	BL4AA	BL6AA	BL6AA	XY, Euploid	XY, Euploid	XY, Euploid	4
ZF‐1	38	IVF	BL4AB	BL5AA	BL5AA	XY, High mosaic trisomy 19	XY, Euploid	XY, Trisomy 19	3
ZF‐2	40	Rescue ICSI	BL4BB	BL5AA	BL5AA	XY, Trisomy 14 and 16	XY, −2p, +14, +16	XY, Trisomy 14 and 16	2
ZF‐3	36	IVF	BL4AB	BL4AB	BL4AB	XY, Euploid	XY, +2, +4, +18, −20	XY, Euploid	3
ZF‐4	40	Rescue ICSI	BL4BA	BL4BA	BL4BA	XY, Euploid	XY, Euploid	XY, Euploid	2
ZF‐5	38	ICSI	BL3AA	BL4AA	BL4AB	XX, Complex aneuploid Monosomy 14 and 16, Partial monosomy 2q35q37.3 (23 Mb)	XX, −2q, −14, −16	XX, Complex aneuploid Monosomy 14 and 16, Partial monosomy 2q35q37.3 (23 Mb)	2
ZF‐6	29	ICSI	BL5AA	BL6AA	BL6AB	XY, Euploid	XY, Euploid	XY, Euploid	0
ZF‐7	29	ICSI	BL4AA	BL4AA	BL4AB	XY, Euploid	XY, Euploid	XY, Euploid	2
ZF‐8	29	ICSI	BL4BA	BL5AA	BL5AB	XY, Euploid	XY, −7, +8	XY, Euploid	2
ZF‐9	38	ICSI	BL4AA	BL5AA	BL5AB	XY, Euploid	XY, Euploid	XY, Euploid	0
ZF‐10	38	ICSI	BL4AA	BL5AA	BL5AB	XX, Trisomy 18 and 21	XX, Non‐info	XX, Trisomy 18 and 21	2
BL2‐1	37	IVF	BL2	BL4AA	BL4AA	XY, Trisomy 10 and 21	XY, Non‐info	XY, Trisomy 10 and 21	1
BL2‐2	39	IVF	BL2	BL3BB	BL3BB	XX, High mosaic monosomy 22	XX, −22	XX, Monosomy 22	3
BL2‐3	36	Rescue ICSI	BL2	BL3BB	BL3BB	XY, Monosomy 16	XY, −16	XY, Monosomy 16	2
BL2‐4	38	IVF	BL2	BL3AC	BL3AC	XY, Trisomy 16	XY, Non‐info	XY, Trisomy 16 and 20	2
BL2‐5	37	IVF	BL2	BL4BB	BL4BB	XX, Euploid	XX, Euploid	XX, Euploid	3
BL2‐6	36	ICSI	BL2	BL4BB	BL4BB	XX, Euploid	XX, −13	XX, Euploid	4
BL2‐7	40	IVF	BL2	BL5AA	BL5AA	XX, Euploid	XX, Euploid	XX, Euploid	3
BL2‐8	37	IVF	BL2	BL4AB	BL4AB	XX, Aneuploid Monosomy 18	XX, Non‐info	XX, Aneuploid Monosomy 18	2
BL2‐9	40	IVF	BL2	BL6AA	BL6AA	XX, Euploid	XX, +3q	XX, Euploid	2
BL2‐10	38	IVF	BL2	BL4BB	BL4BB	XX, Euploid	XX, Non‐info	XX, Euploid	1

Abbreviations: BL2, early blastocysts at day 4; ICSI, intracytoplasmic sperm injection; IVF, *in‐vitro* fertilization; SCM, spent culture medium; TE, trophectoderm; WE, whole embryo; ZF, zona pellucida free.

For the 35 blastocysts analyzed, the karyotypes of the WE, SCM, and TE were 100% (35/35), 80% (28/35), and 100% (35/35), respectively. The concordance rates for the WE versus the SCM, the WE versus the TE, and the TE versus the SCM were 51.4% (18/35), 91.4% (32/35), and 51.4% (18/35), respectively (Table S1). The number of contractions was 2.3 ± 1.5 (Table S1). The aneuploid rates of the WE, TE, and SCM for all embryos were 42.9% (15/35), 45.7% (16/35), and 45.7% (16/35), respectively (Table S1). Non‐information or no DNA was observed in 20.0% (7/35) of patients with SCM.

### 3.2 Culture times of the SCM

Fifteen thawed d5‐BLs were cultured for 8, 16, and 24 h and observed over time (*n* = 5 for each). The concordance rates of the WE versus the SCM, the WE versus the TE, and the TE versus the SCM for all SCM culture times were 60.0% (9/15), 93.3% (14/15), and 53.3% (8/15), respectively (Table S1). The number of contractions was 2.7 ± 1.9 (Table S1). The aneuploid rate in the WEs was 40.0% (6/15) (Table S2), and the karyotype of the aneuploid was low mosaic (Table 1). The aneuploid rate in the TE was 46.7% (7/15) (Table S1), and the karyotypes of the aneuploids were low mosaic and complex mosaic (Table 1). The aneuploidy rate in the SCM was 53.3% (8/15), the rate of no DNA and non‐information was 13.3% (2/15) (Table 1), and one of the aneuploids had a low mosaic rate (Table 1).

**FIGURE 3:**
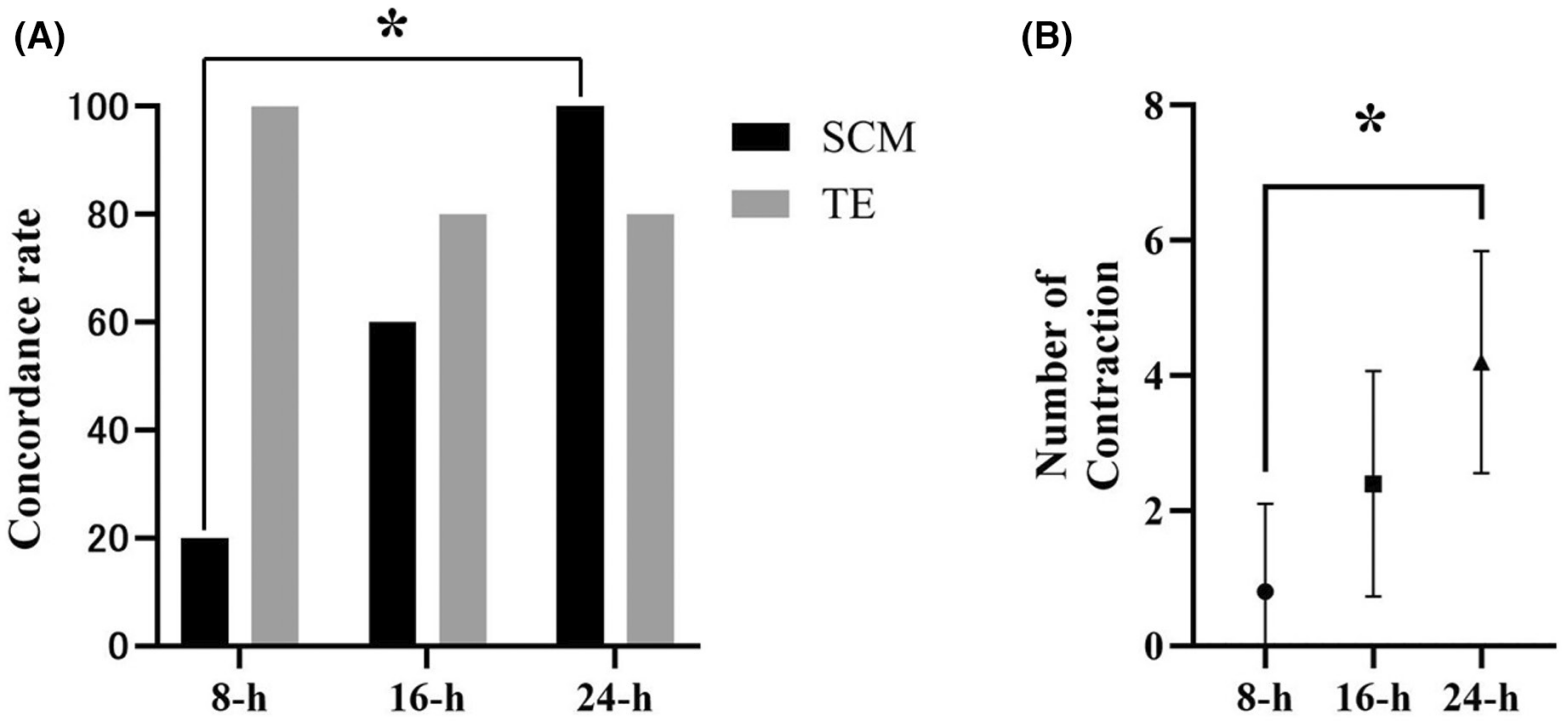
Concordance rate and number of contractions in the culture time. (A) Concordance rate with WE at each culture time. (B) Number of contractions at each culture time. Statistical significance is indicated by the Dunn's multiple comparison test. Significant difference between groups, *p < 0.05.

The concordance of the karyotypes of the WE, SCM, and TE at each culture time point was examined. Only results that were identical with respect to euploidy and degree of mosaicism were considered concordant; partial concordance was considered discordant. The SCM for 16 and 24 h was successfully analyzed in all cases (Table 1). The SCM for 8 h was analyzed in 60.0% (3/5) of the cases, the remainder of which consisted of non‐information or no DNA (Table 1). At 8, 16, and 24 h, the concordance rate between the WE versus the SCM was 20% (1/5), 60.0% (3/5), and 100% (5/5), respectively (Table S2), with significant differences between 8 and 24 h (Figure 3A). Two samples showed partial concordance at 8 h (8–2 and 8–4), whereas two samples showed complete disagreement at 16 h (16–2 and 16–5) (Table 1). For the WE and SCM, the concordance rate for each culture period was high, ranging from 80 to 100%. At 8, 16, and 24 h, the concordance rate between the TE versus the SCM was 20% (1/5), 40.0% (2/5), and 80% (5/5), respectively, with no significant differences (Table S2). For each SCM culture time point, 24 h was found to be optimal.

Next, considering that the number of contractions is important in increasing the concordance rate, we examined the number of contractions at each culture time. At 8, 16, and 24 h, the number of contractions was 0.8 ± 1.1, 3.0 ± 0.9, and 4.4 ± 1.3, respectively (Table S2), and there were significantly more contractions at 24 h than at 8 (Figure 3B).

### 3.3 Influence of the presence or absence of the ZP

After thawing and removal of the ZP, d5‐BLs were analyzed for WE, SCM, and TE karyotypes after culturing under time‐lapse for 24 h (*n* = 10). ZF blastocysts were cultured at the 24‐h culture time that was determined to be optimal, and the results of blastocytes with the ZP (ZP+) cultured for the same 24 h were used for comparison. The aneuploid rates of the WE, TE, and SCM for all embryos were 40.0% (4/10) each (Table S3).

**FIGURE 4:**
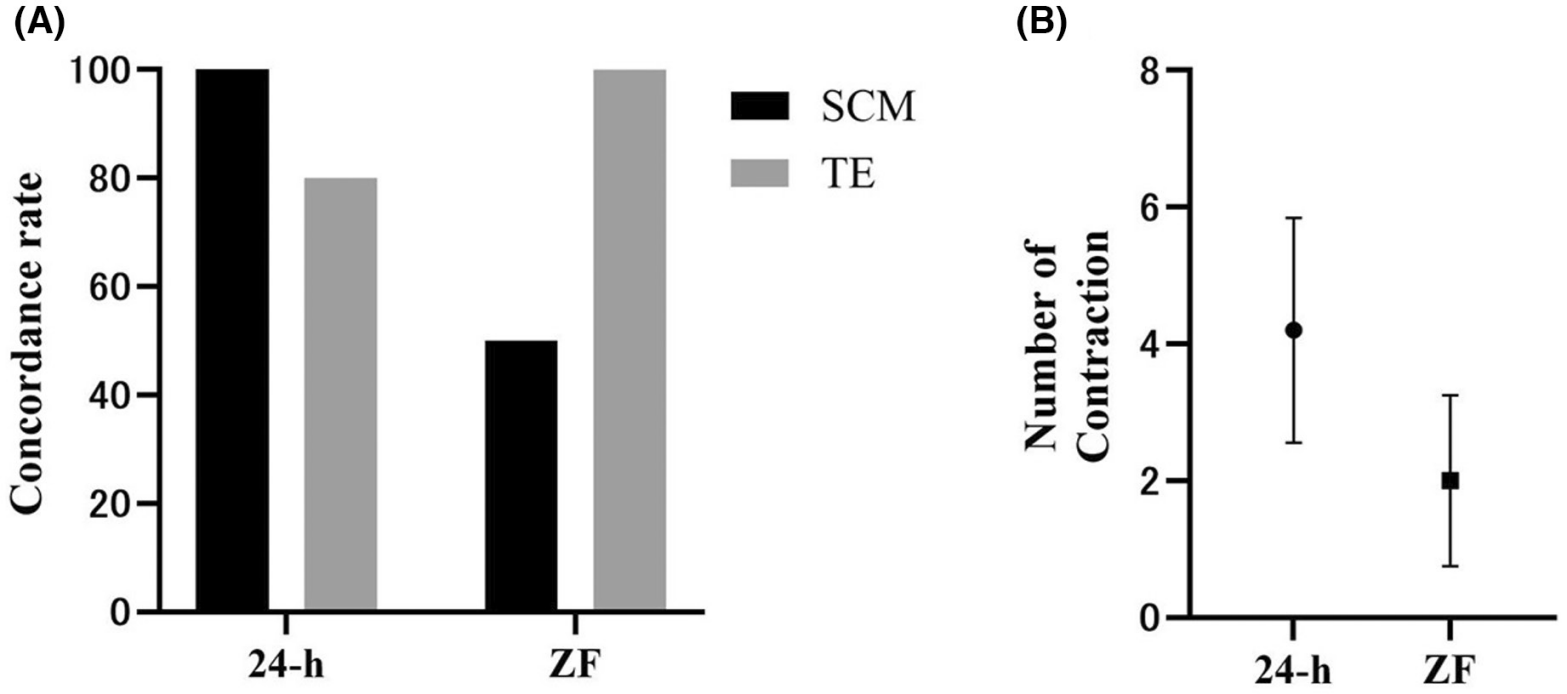
Concordance rate and number of contractions with and without zona pellucida (ZP). (A) Concordance rate for whole embryo (WE) with and without ZP. (B) Number of contractions with and without ZP. ZF, day 5 blastocysts are thawed and zona pellucida is removed and biopsied after culture for 24 h. Statistical significance is indicated by the Dunn's multiple comparison test. No difference between groups.

Next, the karyotype concordance rates for the WE, SCM, and TE were examined. The concordance rates for the WE versus the SCM, the WE versus the TE, and the TE versus the SCM were 50.0% (5/10), 100.0% (10/10), and 50.0% (5/10), respectively (Table S3). The five concordances in the SCM were euploid (ZF‐4, ZF‐6, ZF‐7, and ZF‐9) and aneuploid (ZF‐5) (Table 1). The five discrepancies were aneuploid‐to‐euploid (ZF‐1), partial aneuploid agreement (ZF‐2), euploid‐to‐aneuploid (ZF‐3 and ZF‐8), and aneuploid‐to‐non‐information (ZF‐10) (Table 1). No significant differences were observed between ZP+ and ZF blastocysts, but a trend toward lower concordance was observed in ZF blastocysts (Figure 4A). The number of contractions was 1.8 ± 1.0, with no significant difference between ZP+ and ZF blastocysts (Figure 4B).

### 3.4 Early blastocysts

d4‐BL2s were thawed. After they were cultured for 24 h under time‐lapse conditions, the karyotypes of the WE, SCM, and TE were analyzed by NGS (*n* = 10). The WE, SCM, and TE values in the d4‐BL2 aneuploid group were 50.0% (5/10), 40.0% (4/10), and 50.0% (5/10), respectively (Table S4).

**FIGURE 5:**
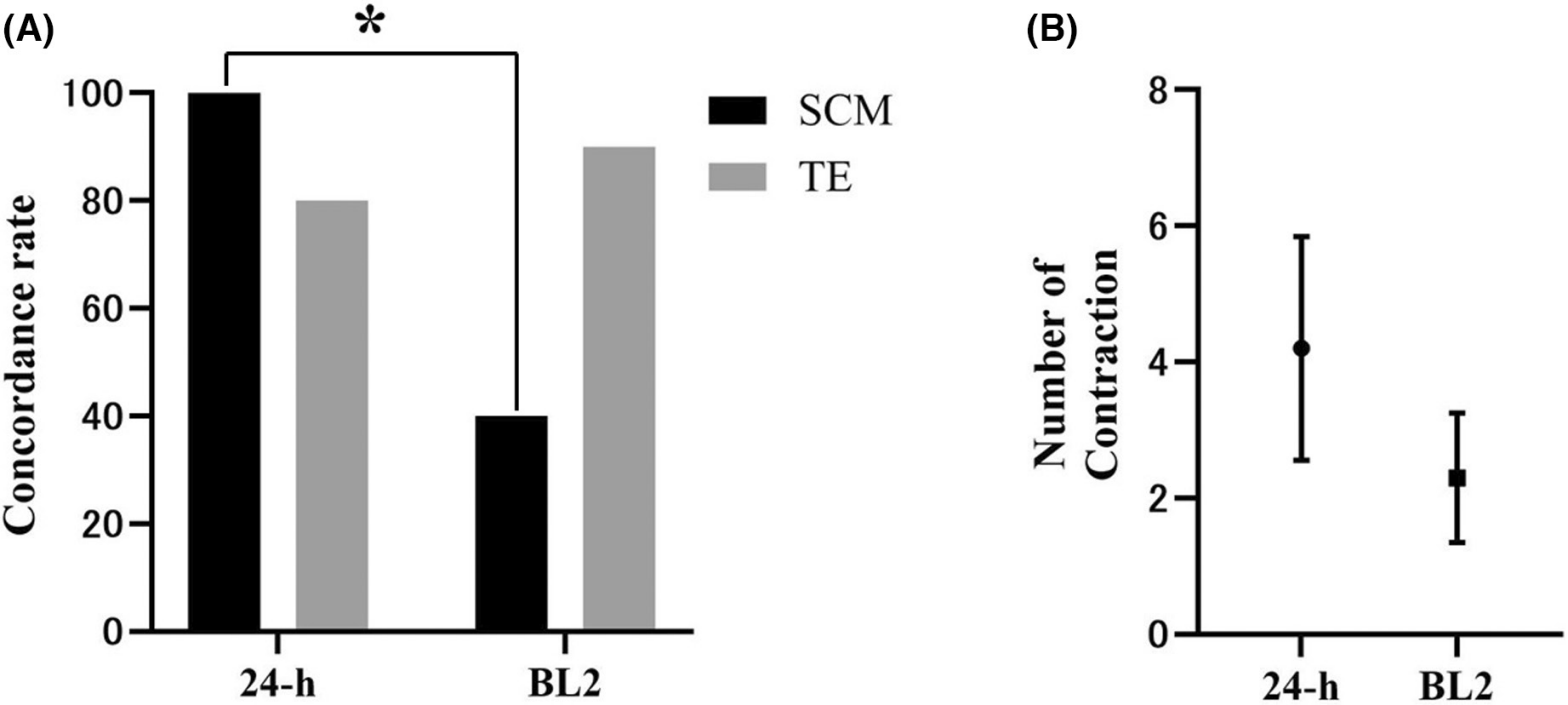
Concordance rate and number of contractions blastocysts transferred on day 4 (d4‐BL2). (A) Concordance rate for whole embryo (WE) in d5‐BL and d4‐BL2. (B) Number of contractions in d5‐BL and d4‐BL2. BL2, early blastocysts on day 4 are thawed and biopsied after culture for 24 h. Statistical significance is indicated by the Dunn's multiple comparison test. Significant difference between groups, *p = 0.044.

Next, we examined karyotype concordance rates for the WE, SCM, and TE. The concordance rates for the WE versus the SCM, the WE versus the TE, and the TE versus the SCM were 40.0% (4/10), 90.0% (9/10), and 40.0% (4/10), respectively (Table S4). In the SCM, two of the four concordances were euploid (BL2‐5 and BL2‐7) and two were aneuploid (BL2‐2 and BL2‐3) (Table 1). There were two discrepancies, with euploid being determined to be aneuploid (BL2‐6 and BL2‐9) (Table 1). Four cases of non‐information were evaluated using strict criteria for median absolute pairwise difference (MAPD) values (MAPD = 0.25). Thus, when the criteria were relaxed, BL2‐1, BL2‐4, BL2‐8, and BL2‐10 were trisomies 10 and 21, trisomies 16 and 18, trisomies 3 and 4, and euploid, respectively (Figure S2). BL2‐1 and BL2‐10 were concordant, whereas BL2‐4 and BL2‐8 were discordant. In the concordance rates of the WE versus the SCM, there was a significant difference between ZP+ blastocysts cultured for 24 h and BL2s (10 cases) (Figure 5A), but not when non‐information was excluded (Table S5). The number of contractions was 2.3 ± 0.9 (Table S4), with no significant difference between ZP+ blastocysts cultured for 24 h and BL2s (Figure 5B).

## 4 DISCUSSION

This study attempted to evaluate the genetic testing performance of WE, SCM, and TE biopsy samples when using d4‐BL2s, with the aim of applying this to embryo transfer using d5‐BLs, which are considered to have the highest pregnancy rate. First, using the d5‐BLs, concordance rates for the 8‐, 16‐, and 24‐h SCM versus the WE and TE versus the WE were compared, and the 24‐h SCM was found to have a higher match rate than the TE. Next, to examine the effect of the presence or absence of the ZP, we compared the agreement rates between the SCM with and without the ZP. These results indicated that the ZP is important for improving the accuracy of niPGT‐A because the SCM without the ZP decreases the agreement rate. Finally, we compared the results of the SCM, TE, and WE of d4‐BL2s for 24‐h culture and found that the TE showed a high agreement rate of 90%, while the SCM showed an overall agreement rate of 40% and a non‐information rate of 40% (even when non‐information was excluded, the agreement rate was 66.7%). In addition, few sex discrepancies were detected (with one showing no DNA in the 8‐h culture SCM).

The origin and developmental mechanism of cfDNA in embryos are not clear but are thought to be derived from programmed cell death, such as apoptosis and necrosis.^43^ Compared to the waveform data obtained by NGS, those obtained from the 8‐ and 16‐h cultures were more unstable than those obtained from the 24‐h cultures (Figures S1–S3). This may be owing to the non‐information and absence of DNA in the SCM with the shortest culture time of 8 h and the low amount of cfDNA in the embryo culture medium. Non‐information or no DNA was not observed after prolonged culture (16 h or later), and a 100% concordance rate was obtained after 24 h of culture. Blastocysts undergo repeated expansion and contraction before hatching and exiting the ZP and are thought to exchange luminal fluid through contraction and expansion.^44^, ^45^ We hypothesized that the cfDNA in the blastocyst cavity generated during culture was expelled from the blastocyst by contraction and expansion and compared the number of contractions at each culture time. The 24‐h culture resulted in significantly more contractions and the highest concordance rate compared with the 8‐h culture. It has recently been reported that 24 h is the optimal incubation time during niPGT‐A, and our results were similar.^46^ However, the mechanism by which cfDNA content increases during contraction and dilation is unknown.

Although the maintenance of intercellular structures is an important role of the ZP,^47^ human oocytes that are ZF develop to the blastocyst stage, and healthy babies are born after embryo transfer.^48^ Therefore, the ZP is not essential for postfertilization development. To investigate whether the ZP inhibits the dispersion of cfDNA into the SCM, the karyotype of blastocysts from which the ZP was removed prior to culture was evaluated by NGS. The results showed that the agreement between the WE and TE was 100%, whereas that between the WE and SCM was 40%. In addition, ZF blastocysts tended to have a lower shrinkage frequency. It has been reported that maternal cell contamination is reduced and the concordance rate increases significantly when cultured in multiple washes rather than in a one‐step wash for niPGT‐A,^49^ and in this study, blastocysts were washed three to four times from ZF blastocysts to culture in order to increase the concordance rate for niPGT‐A. In addition, it has been reported that being ZF reduces cytoplasmic fragmentation in time‐lapse analyses of human embryos.^50^ Therefore, it is possible that the reduction in cytoplasmic fragmentation by ZF‐suppressed programmed cell death is a factor in the generation of cfDNA. Furthermore, the fact that blastocysts in the ZP state were washed multiple times, which did not ensure a sufficient amount of cfDNA, may have reduced the concordance rate.

Embryo transfer is performed on d5‐BLs at many centers, as d5‐BLs have the highest clinical pregnancy rate. We considered the clinical application of niPGT‐A and evaluated its concordance rate in SCMs with d4‐BL2s cultured for 24 h. WE and SCM concordance for d4‐BL2s was 40%, a significant difference from d5‐BLs cultured for 24 h. However, when non‐information in four cases was excluded in the SCM of d4‐BL2s, the concordance rate was 66.7%, and there was no significant difference between this SCM and the d5‐BLs cultured for 24 h (Table S4). Furthermore, because a strict MAPD value threshold for non‐information was set in this study for MAPD values exceeding 0.25, we examined whether the agreement rate increased when the threshold was relaxed. The concordance rate was as low as 50% (two out of four concordant cases), and no significant difference was found between the d5‐BLs after 24 h of culture (data not shown). Apoptosis has been reported to occur at a relatively low frequency in early mouse blastocysts and to increase in later stages.^51^ In human blastocysts, apoptosis has also been reported to occur in TE cells during pre‐implantation embryogenesis, playing an important role in normal embryonic development and quality control.^52^ Therefore, in the case of d4‐BLs, apoptosis, which plays an important role in the development of cfDNA, may have occurred at a low frequency for a certain period of time, resulting in a lack of cfDNA and thus a reduced fitness rate. Therefore, SCM techniques for early blastocysts need to be optimized, including extended culture time.

Maternal DNA contamination can adversely affect the niPGT‐A results. niPGT‐A concordance rates are mostly reported for Intracytoplasmic Sperm Injection (ICSI) cycles^25^, ^28^, ^30^, ^42^, ^53^, ^54^; however, maternal genetic contamination remains a problem.^29^ Therefore, it is important to use cryopreserved blastocysts to prevent maternal DNA contamination.^53^ It has also been reported that niPGT‐A in conventional IVF (con‐IVF) cycles can increase the concordance rate by interspersing multiple embryo washes.^49^ In this study, 26 frozen blastocysts con‐IVF and nine ICSI embryos were subjected to multiple washing processes without awareness, and there was almost no evidence of maternal cell contamination. In short, embryo freezing before niPGT‐A and multiple washes just before culture for the SCM are likely to be useful in improving the accuracy of the SCM; however, unnecessary freezing should be avoided in clinical applications, and the protocol of this study needs to be optimized.

Our study had several limitations. First, 35 blastocysts were donated for this study; however, the sample size was small. Therefore, a larger sample size may result in a significant difference in the comparison of concordance rates between ZP+ and ZF blastocysts. The second limitation was the clinical outcome. Mosaic embryos may recover from aneuploidy during postimplantation development,^55^ and it is not known whether the chromosomal state of the implanted embryo is identical to that of the fetus. Third, only frozen embryos were used in this study. The amount of cfDNA increased with increased cell death during the thawing process and subsequent culture process, which may have resulted in more favorable conditions compared to niPGT‐A in fresh embryos. In clinical IVF, unnecessary freezing and thawing should be avoided, and optimization in fresh embryos may be important in the future.

In conclusion, niPGT‐A requires at least 24 h of culture. For the first time to our knowledge, it was found that niPGT‐A showed a higher concordance rate than TE biopsies when collected on day 6 and that 24‐h cultures had the highest number of contractions. This is the first time, to our knowledge, that the ZP has been found to be required for SCM culture. In addition, in niPGT‐A using SCMs after 24 h of culture with d4‐BL2s, the concordance rate for SCMs was lower than that for the TE. In the present study, high concordance rates were observed in almost all specimens used for sex determination. This study may therefore aid in the clinical application of niPGT‐A, which is not yet technically mature.

## CONFLICT OF INTEREST STATEMENT

The authors declare no conflict of interests for this article.

## ETHICS STATEMENT

Ethical approval was obtained from the Clinical Research Ethics Review Committee of Mie University Hospital (H2022‐175), and the study was conducted in accordance with the guidelines established by the Ethics Committee of the Japan Society of Obstetrics and Gynecology (No. 104). All procedures followed were in accordance with the ethical standards of the responsible committee on human experimentation (institutional and national) and with the Helsinki Declaration of 1964 and its later amendments.

## INFORMED CONSENT

Informed consent was obtained from all patients for being included in the study.

## CLINICAL TRIAL REGISTRATION

This study was registered in the Japan Registry of Clinical Trials (jRCT1040220121).

## Supporting information


Appendix S1:

## Data Availability

The data that support the findings of this study are available from the corresponding author, Hiroki Takeuchi, upon reasonable request.
