# Supplementary material for: Conditions for improved accuracy of noninvasive preimplantation genetic testing for aneuploidy: Focusing on the zona pellucida and early blastocysts
Source: Reprod Med Biol. 2024 Sep 10;23(1):e12604. doi: 10.1002/rmb2.12604 (PMC11387587; doi:10.1002/rmb2.12604)
Supplement: Supplementary file 1 — Appendix S1: [file RMB2-23-e12604-s001.zip › rmb212604-sup-0004-TableS1.pdf]

			Total	Culture time
Euploid and Aneuploid rate	WE	Euploid	57.1% (20/35)	60.0% (9/15)
		aneuploid	42.9% (15/35)	40.0% (6/15)
	SCM	Euploid	34.3% (12/35)	33.3% (5/15)
		aneuploid	45.7% (16/35)	53.3% (8/15)
	TE	Euploid	57.3% (19/35)	53.3% (8/15)
		aneuploid	45.7% (16/35)	46.7% (7/15)
Concordance rate	WE vs SCM		51.4% (18/35)	60.0% (9/15)
	WE vs TE		91.4% (32/35)	93.3% (14/15)
	TE vs SCM		51.4% (18/35)	53.3% (8/15)
Number of Contractions			2.3 ± 1.5	2.7 ± 1.9

SCM, spent culture medium; TE, trophectoderm; WE, whole embryo.
