# Supplementary material for: Conditions for improved accuracy of noninvasive preimplantation genetic testing for aneuploidy: Focusing on the zona pellucida and early blastocysts
Source: Reprod Med Biol. 2024 Sep 10;23(1):e12604. doi: 10.1002/rmb2.12604 (PMC11387587; doi:10.1002/rmb2.12604)
Supplement: Supplementary file 1 — Appendix S1: [file RMB2-23-e12604-s001.zip › rmb212604-sup-0005-TableS2.pdf]

			8-h	16-h	24-h
Euploid and Aneuploid rate	WE	Euploid	40.0% (2/5)	0.0% (0/5)	40.0% (2/5)
		aneuploid	60.0% (3/5)	100.0% (5/5)	60.0% (3/5)
	SCM	Euploid	0.0% (0/5)	60.0% (3/5)	40.0% (2/5)
		aneuploid	60.0% (3/5)	40.0% (2/5)	60.0% (3/5)
	TE	Euploid	40.0% (2/5)	80.0% (4/5)	40.0% (2/5)
		aneuploid	60.0% (3/5)	20.0% (1/5)	60.0% (3/5)
Concordance rate	WE vs SCM		20.0% (1/5)	60.0% (3/5)	100% (5/5)
	WE vs TE		100% (5/5)	80.0% (4/5)	80.0% (4/5)
	TE vs SCM		20.0% (1/5)	40.0% (2/5)	80.0% (4/5)
Number of Contractions			0.8 ± 1.1	3.0 ± 0.9	4.4 ± 1.3

SCM, spent culture medium; TE, trophectoderm; WE, whole embryo.
