# Supplementary material for: Conditions for improved accuracy of noninvasive preimplantation genetic testing for aneuploidy: Focusing on the zona pellucida and early blastocysts
Source: Reprod Med Biol. 2024 Sep 10;23(1):e12604. doi: 10.1002/rmb2.12604 (PMC11387587; doi:10.1002/rmb2.12604)
Supplement: Supplementary file 1 — Appendix S1: [file RMB2-23-e12604-s001.zip › rmb212604-sup-0007-TableS4.pdf]

			BL2
Euploid and Aneuploid rate	WE	Euploid	50.0% (5/10)
		aneuploid	50.0% (5/10)
	SCM	Euploid	20.0% (2/10)
		aneuploid	40.0% (4/10)
	TE	Euploid	50.0% (5/10)
		aneuploid	50.0% (5/10)
Concordance rate	WE vs SCM		40.0% (4/10)
	WE vs TE		90.0% (9/10)
	TE vs SCM		40.0% (4/10)
Number of Contractions			2.3 ± 0.9

SCM, spent culture medium; TE, trophectoderm; WE, whole embryo.
