# Supplementary figures and images for: Conditions for improved accuracy of noninvasive preimplantation genetic testing for aneuploidy: Focusing on the zona pellucida and early blastocysts
Source: Reprod Med Biol. 2024 Sep 10;23(1):e12604. doi: 10.1002/rmb2.12604 (PMC11387587; doi:10.1002/rmb2.12604)

8-1

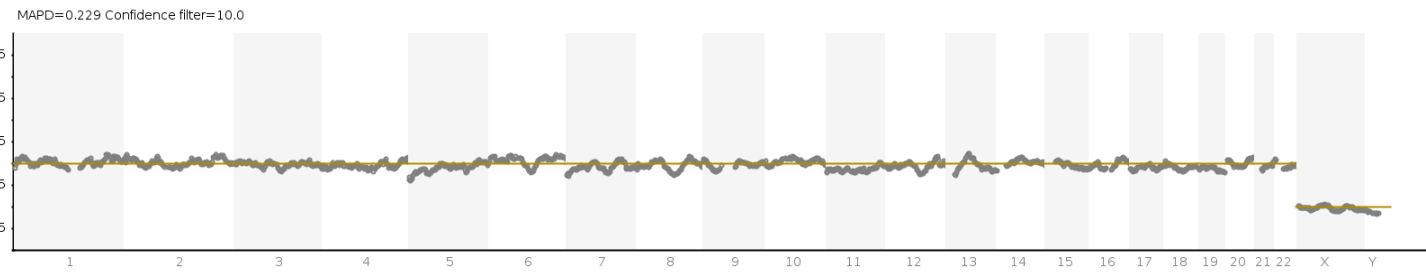

8-2

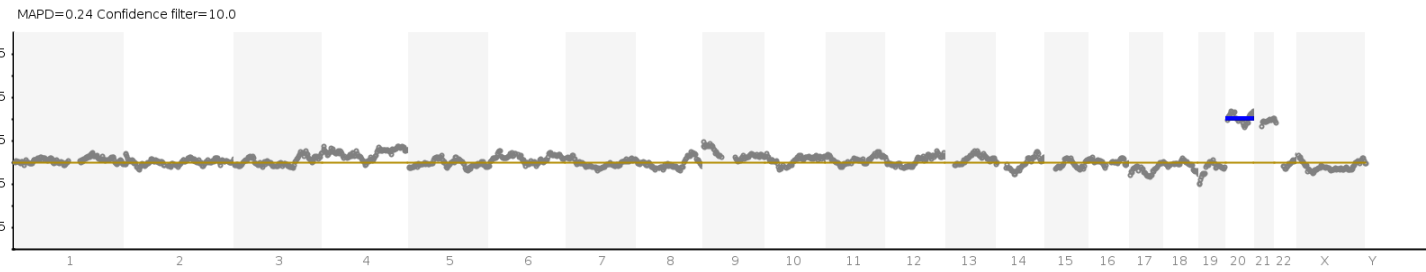

8-3

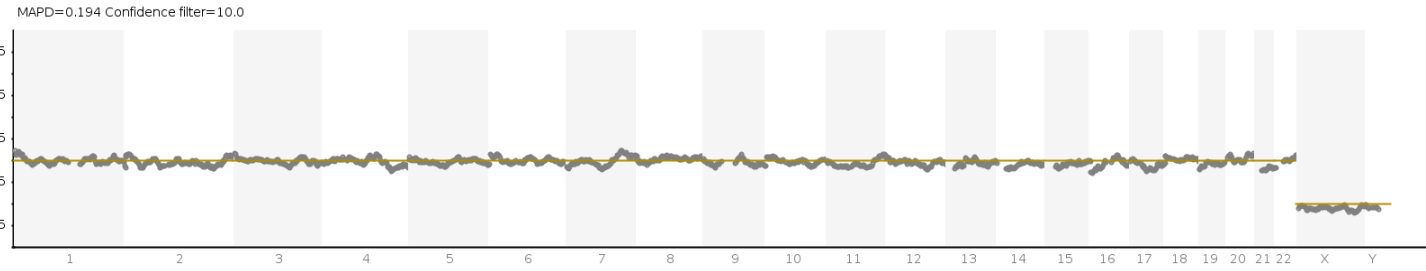

8-4

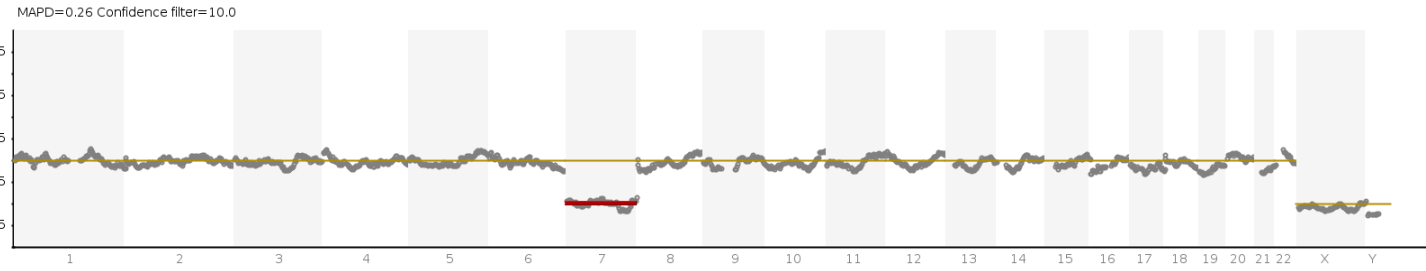

8-5

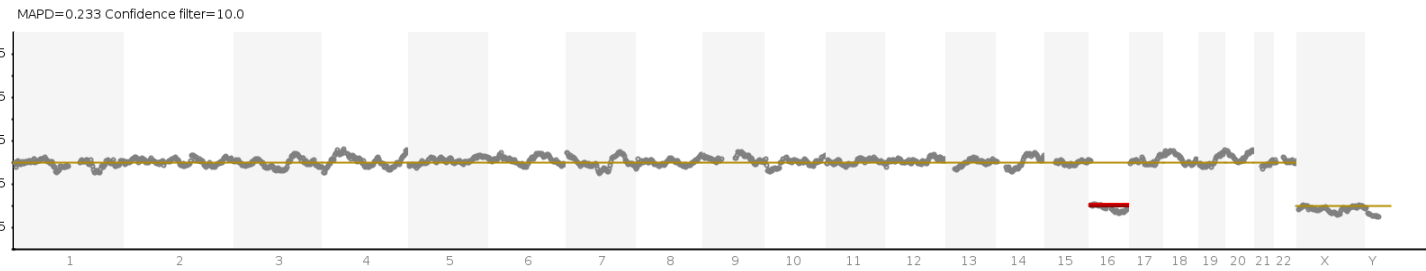

16-1

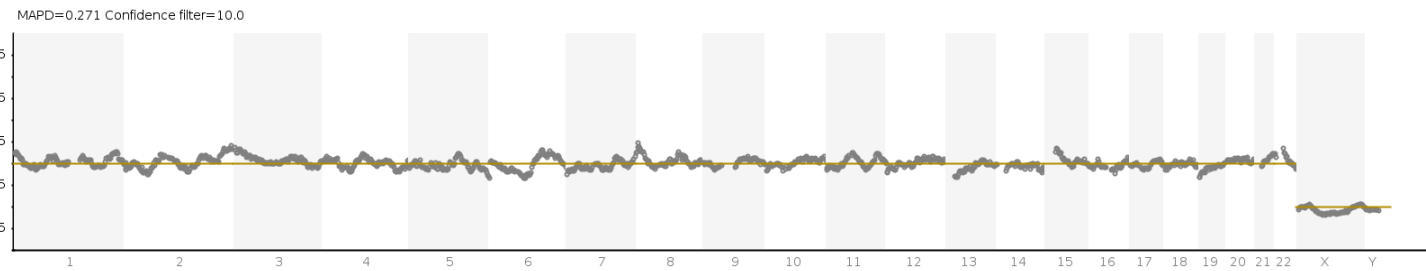

16-2

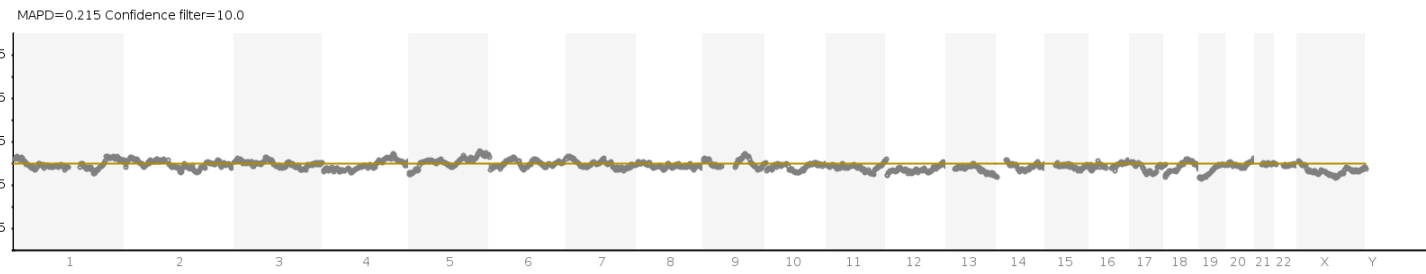

16-3

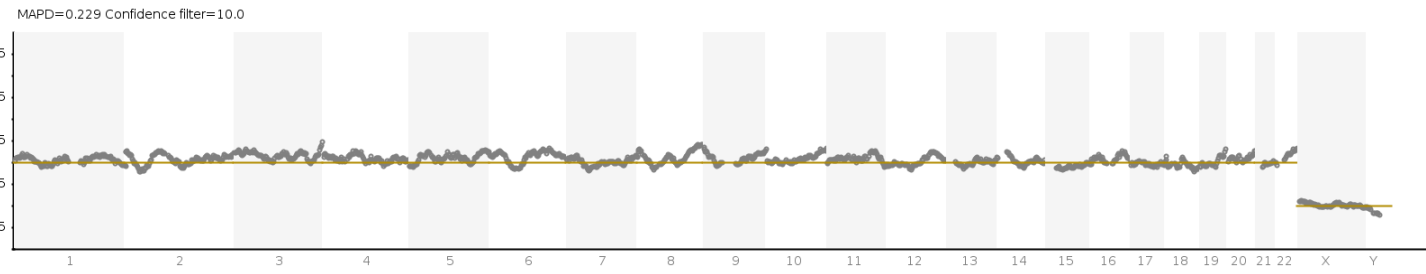

16-4

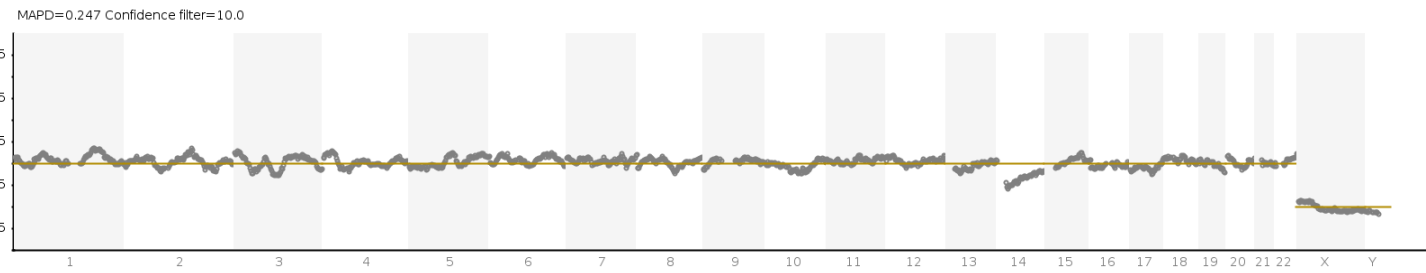

16-5

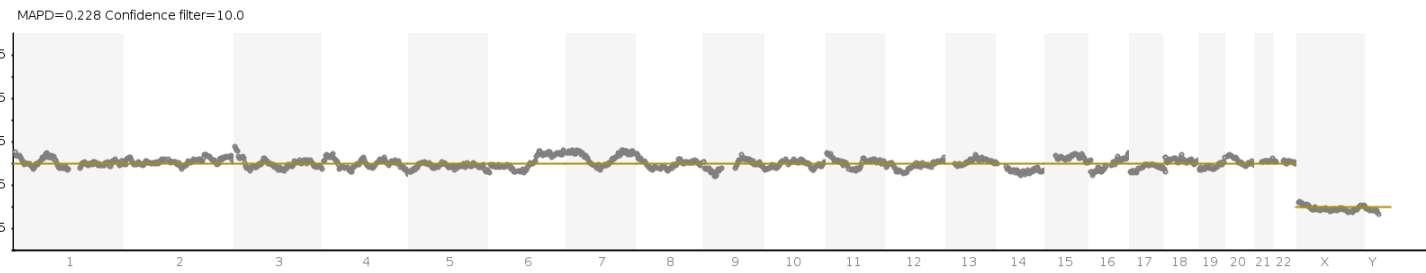

24-1

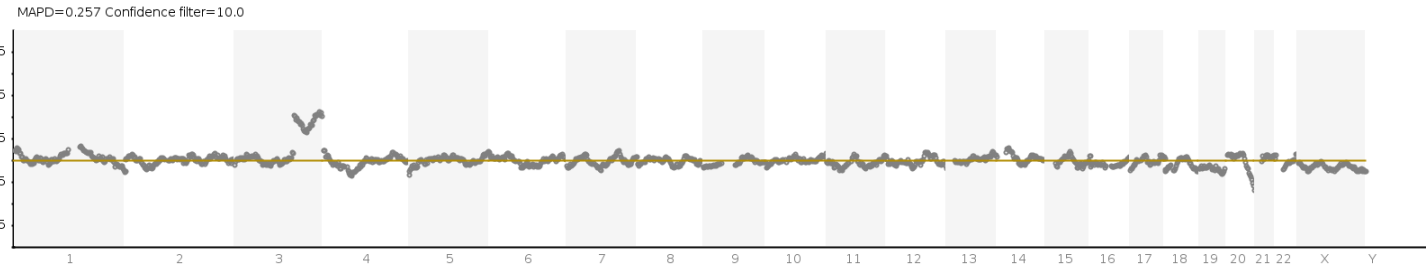

24-2

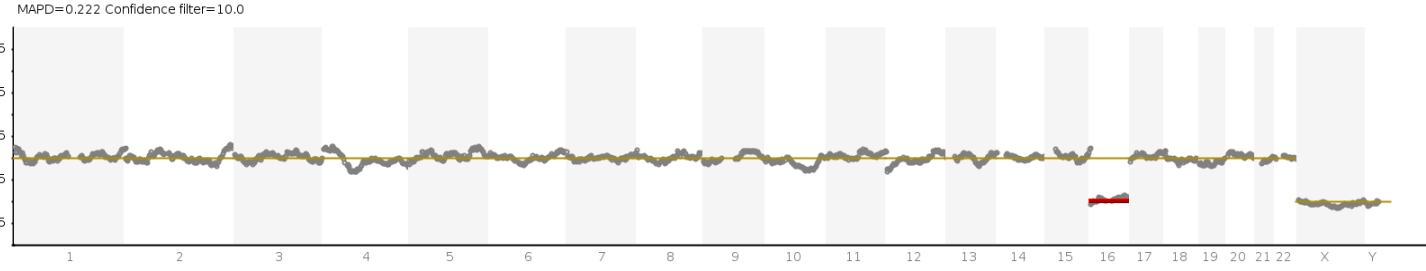

24-3

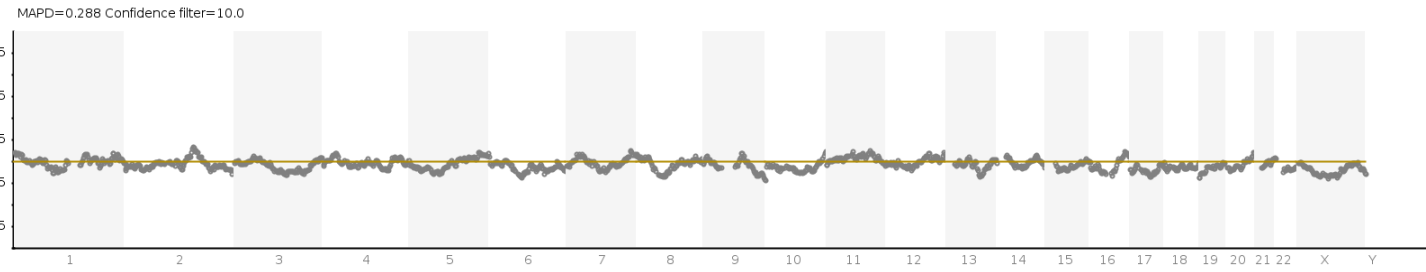

24-4

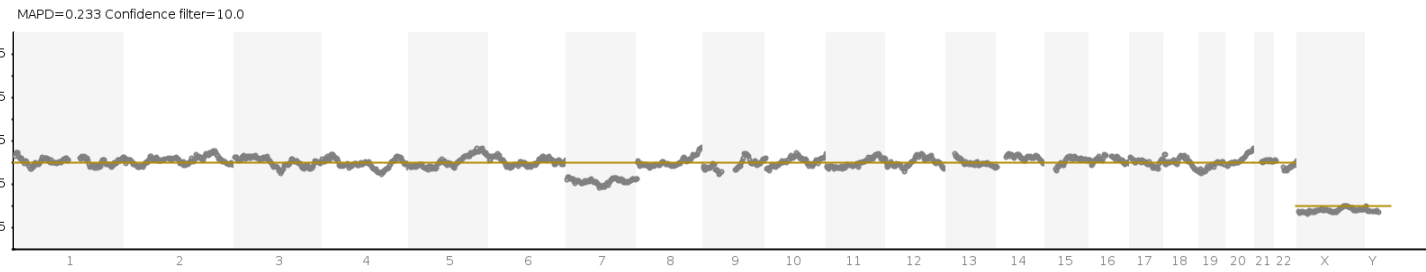

24-5

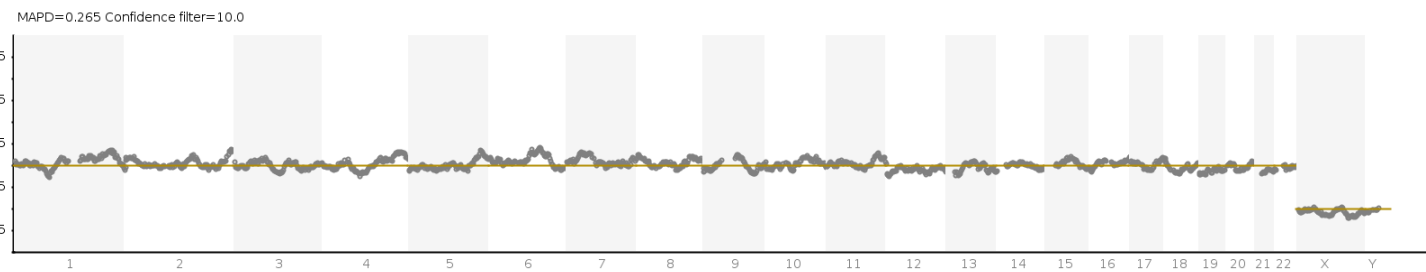

ZF-1

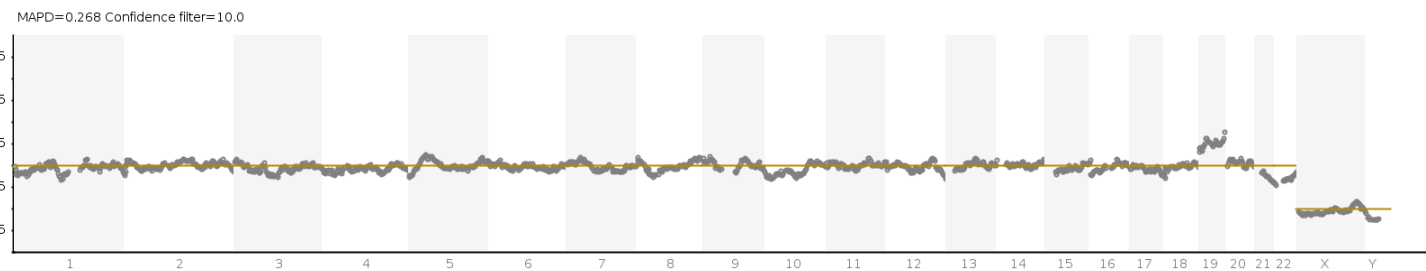

ZF-2

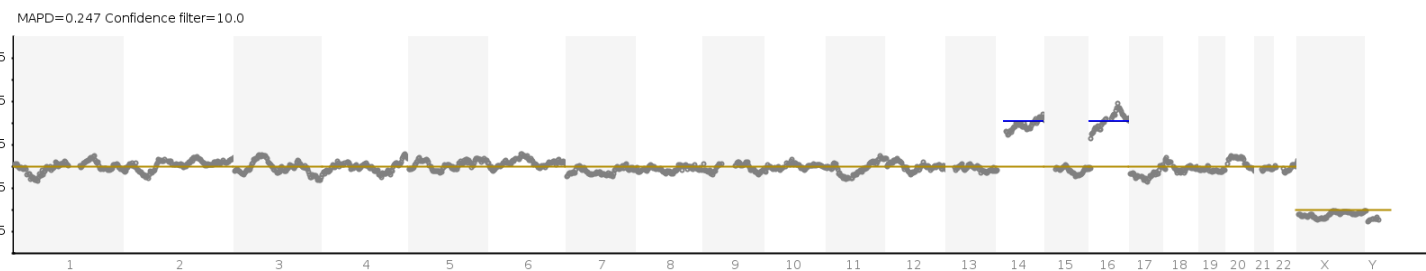

ZF-3

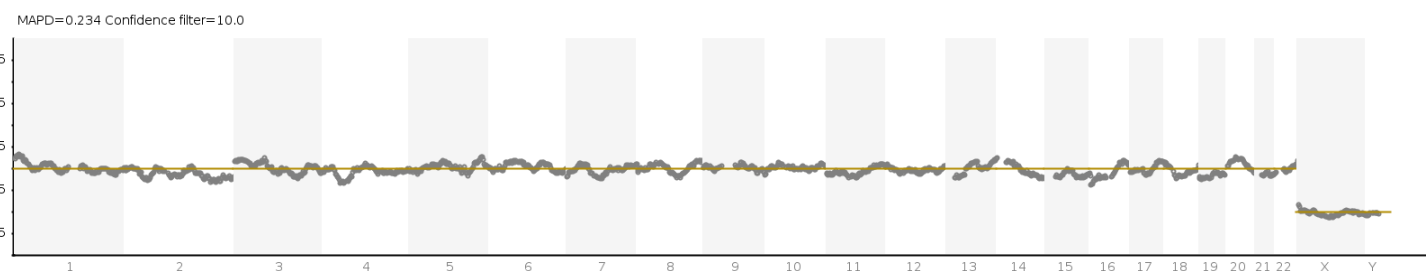

ZF-4

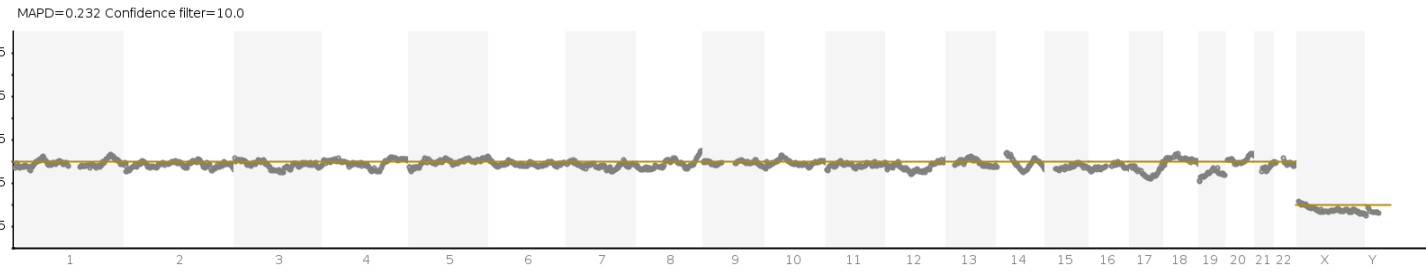

ZF-5

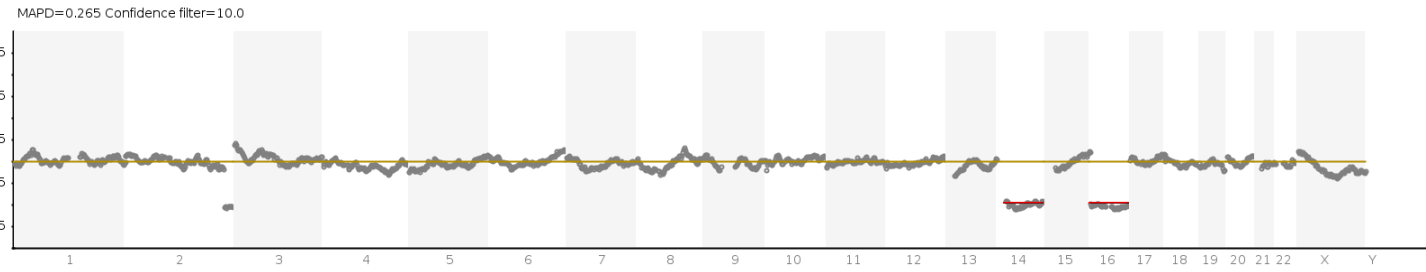

ZF-6

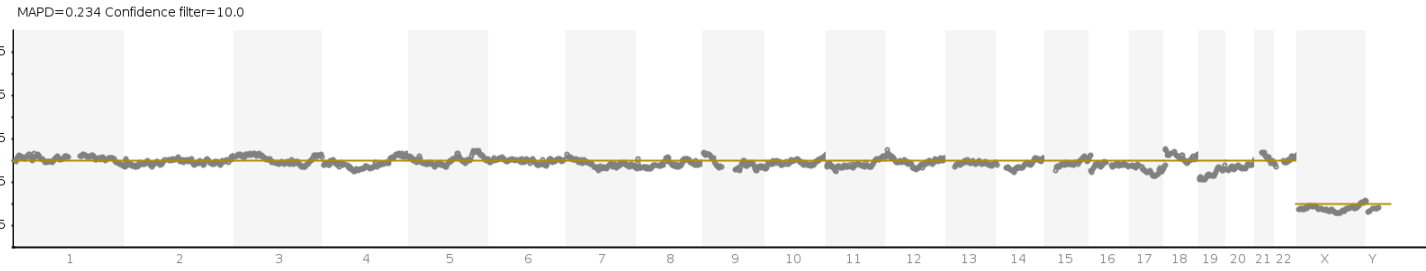

ZF-7

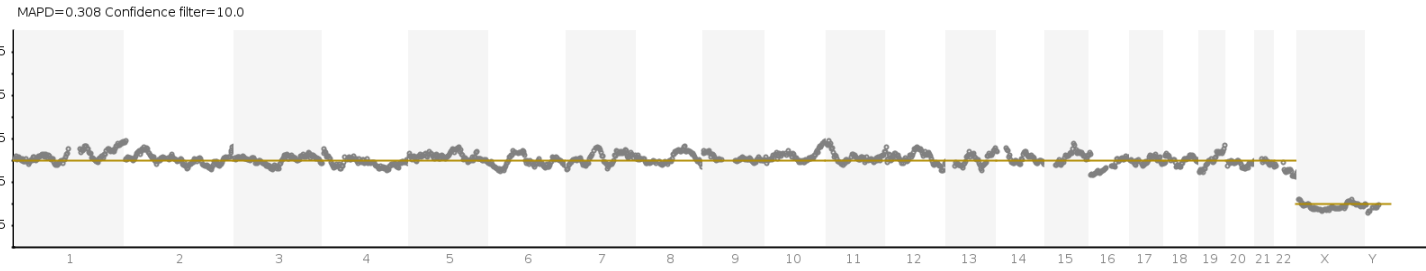

ZF-8

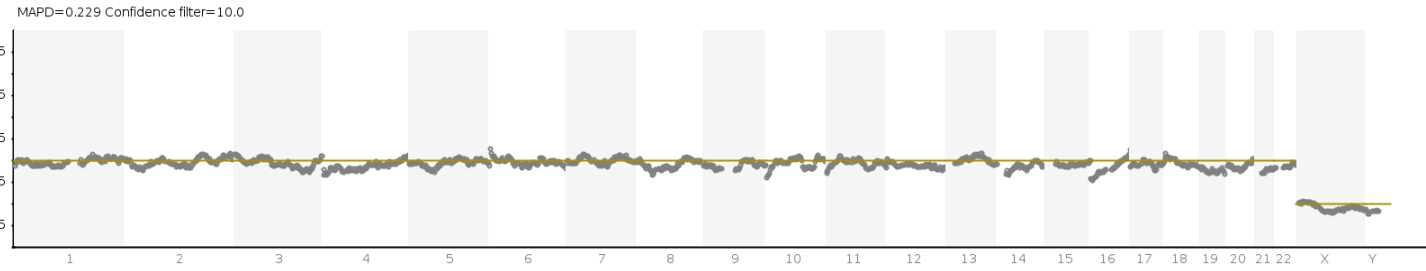

ZF-9

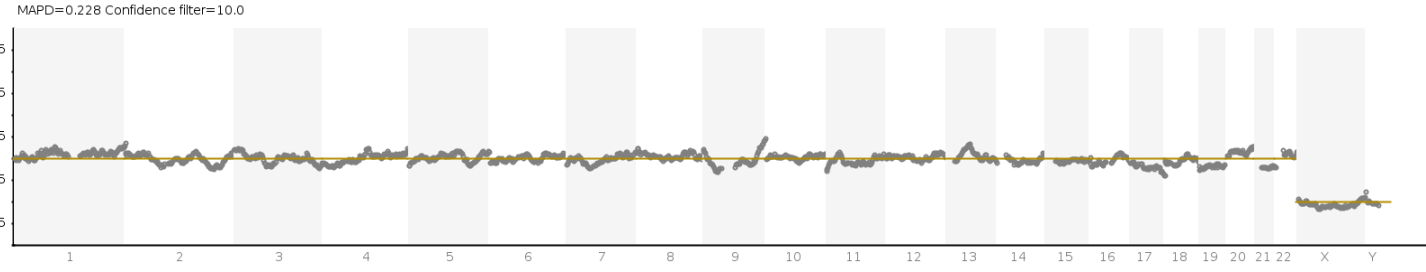

ZF-10

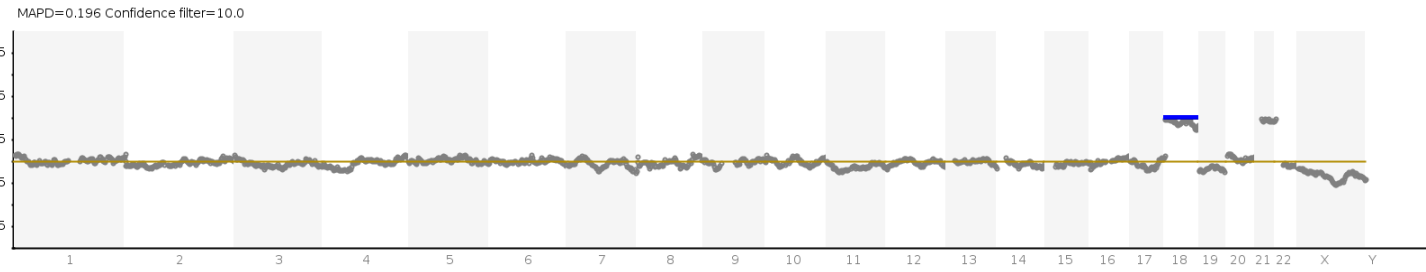

BL2-1

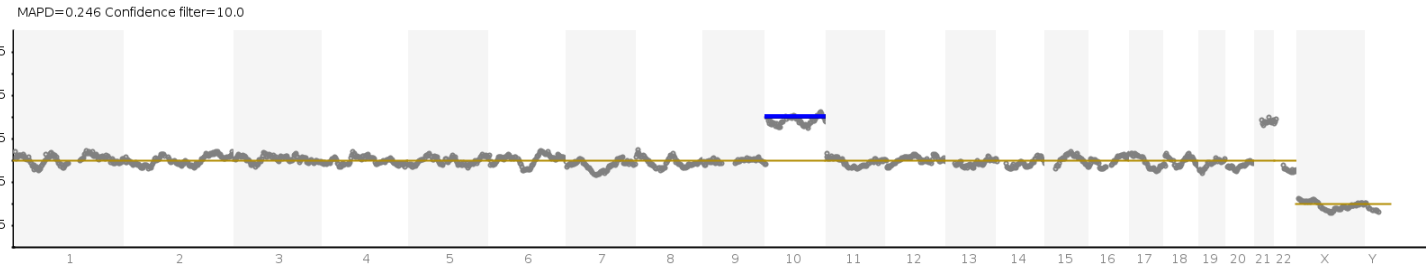

BL2-2

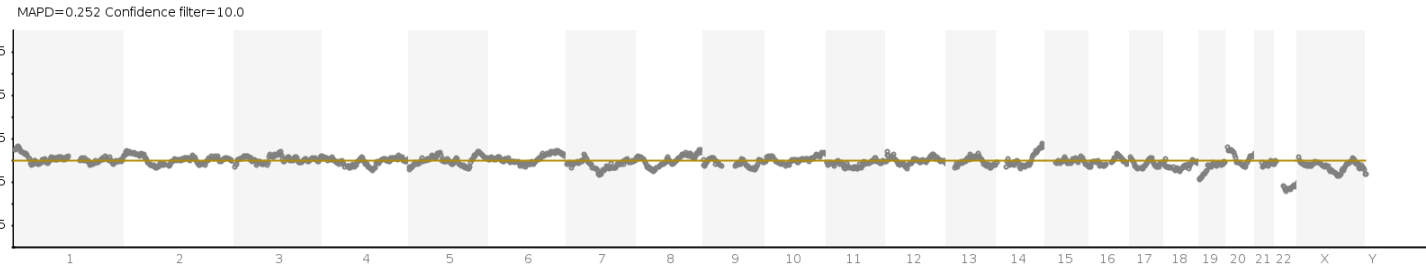

BL2-3

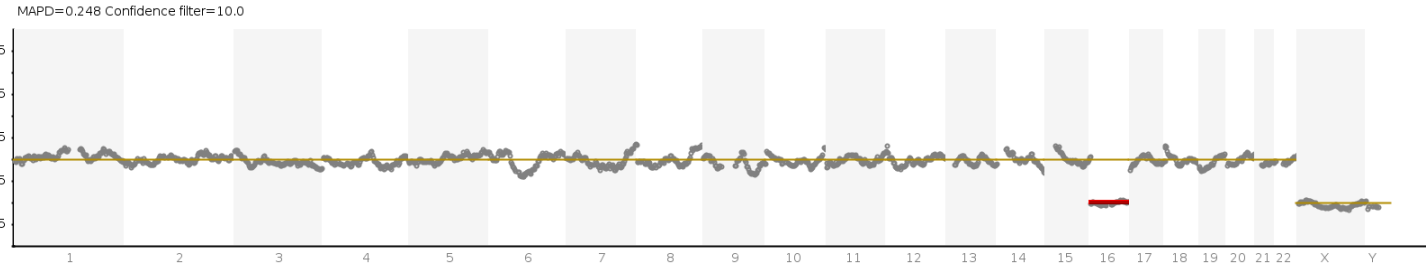

BL2-4

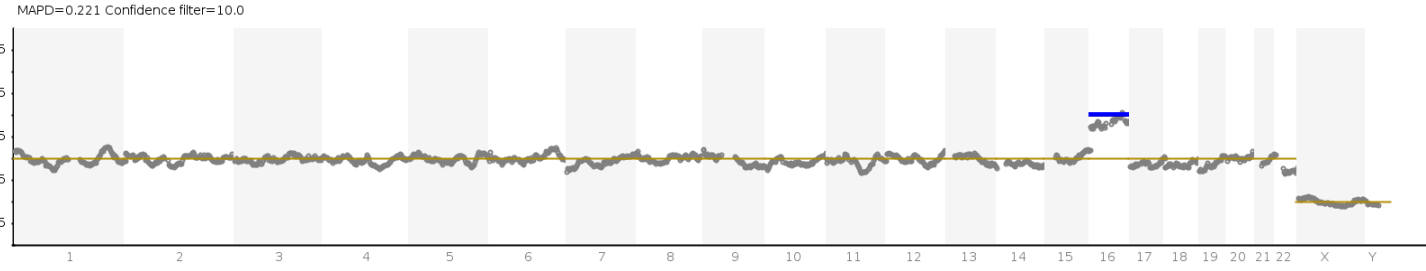

BL2-5

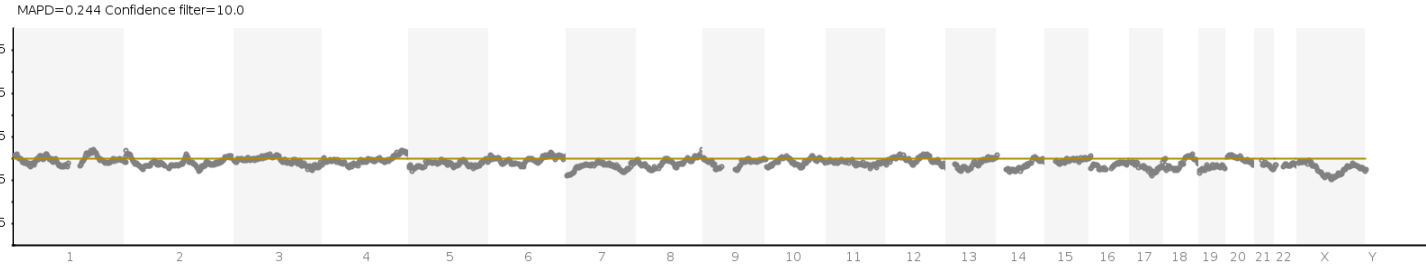

BL2-6

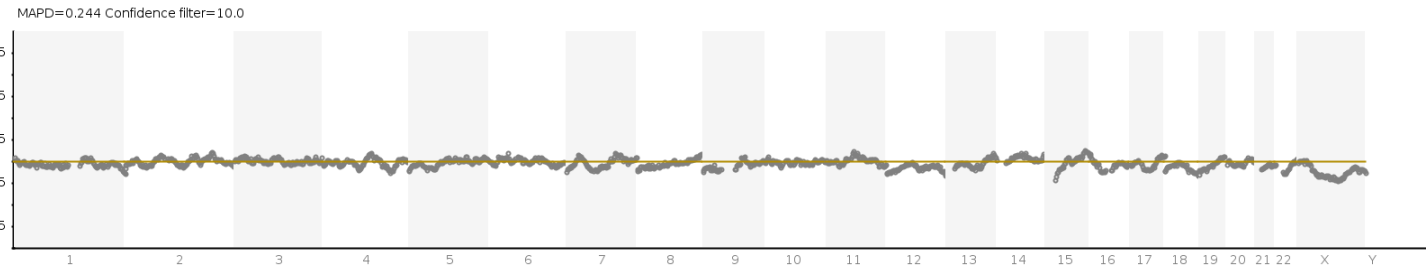

BL2-7

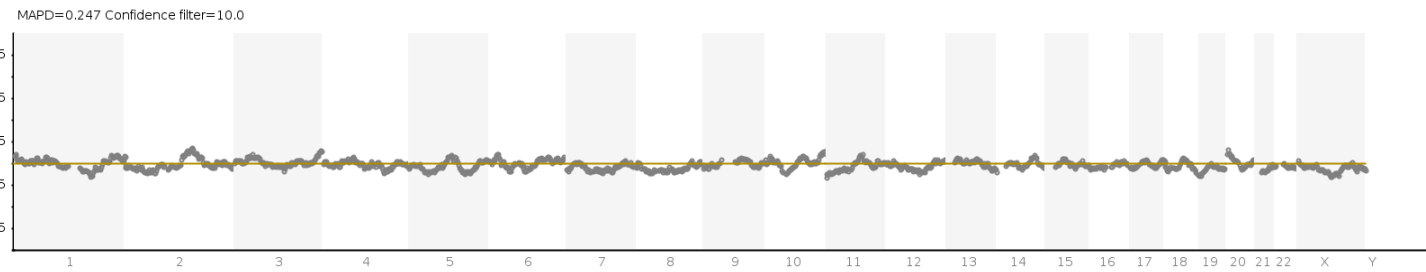

BL2-8

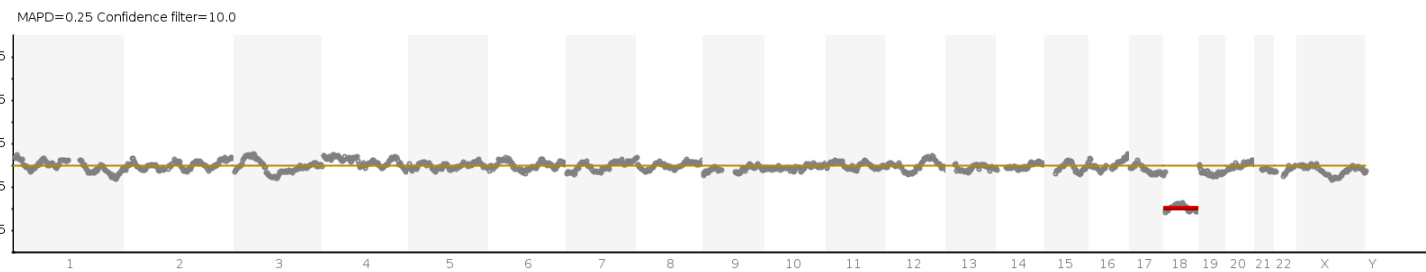

BL2-9

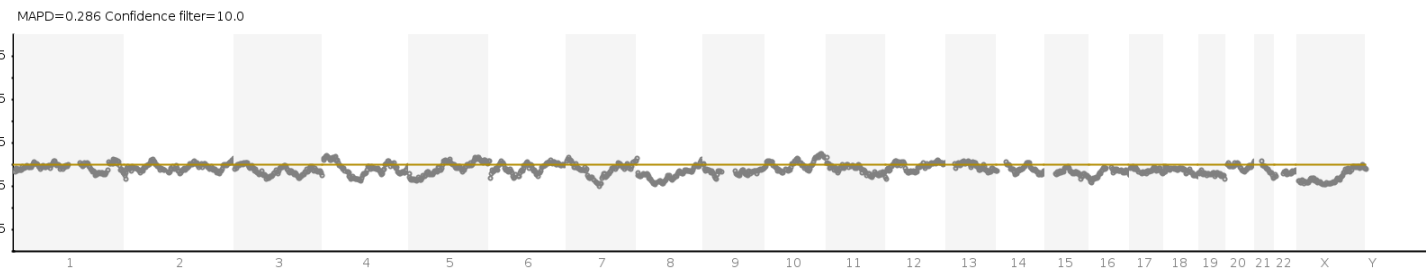

BL2-10

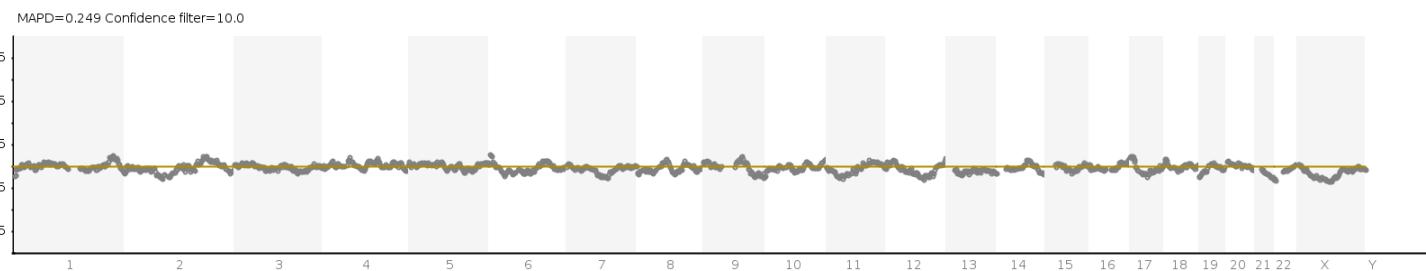

Supplement: Supplementary file 1 — Appendix S1: [file RMB2-23-e12604-s001.zip › rmb212604-sup-0001-FigureS1.pdf]

8-1

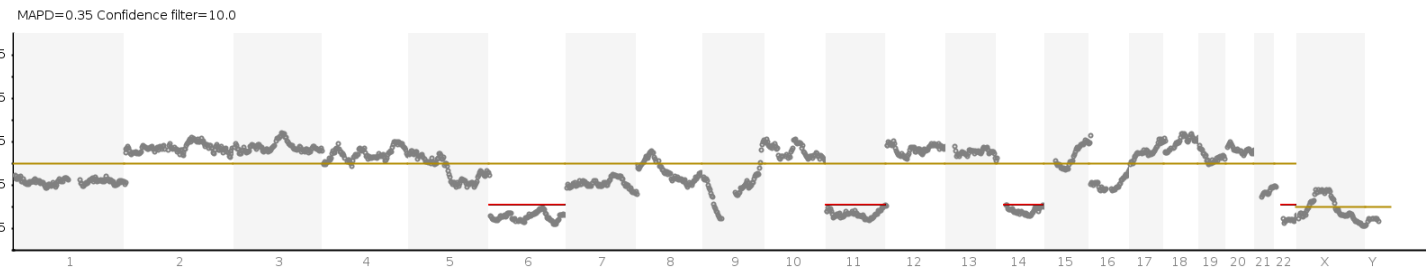

8-2

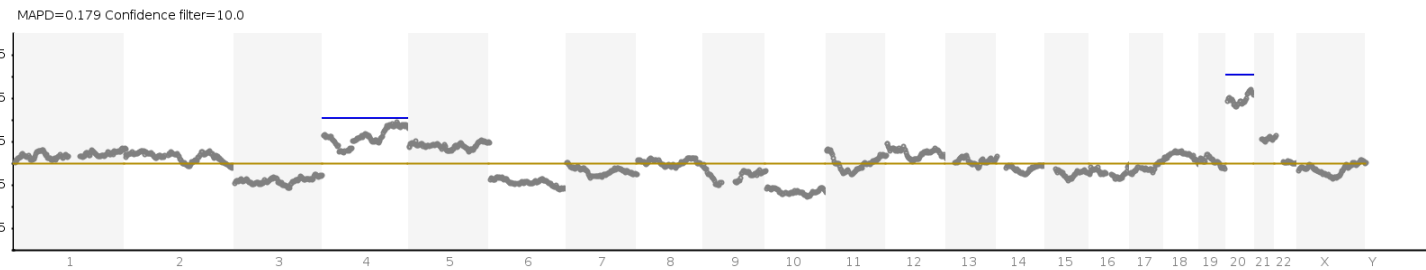

8-3

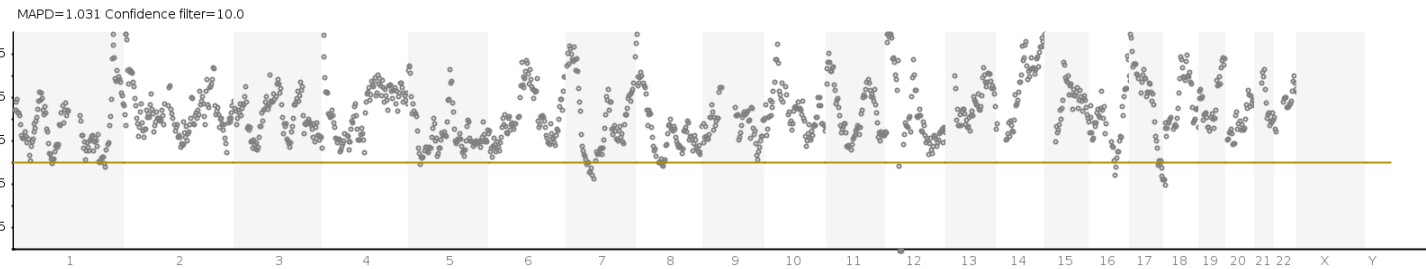

8-4

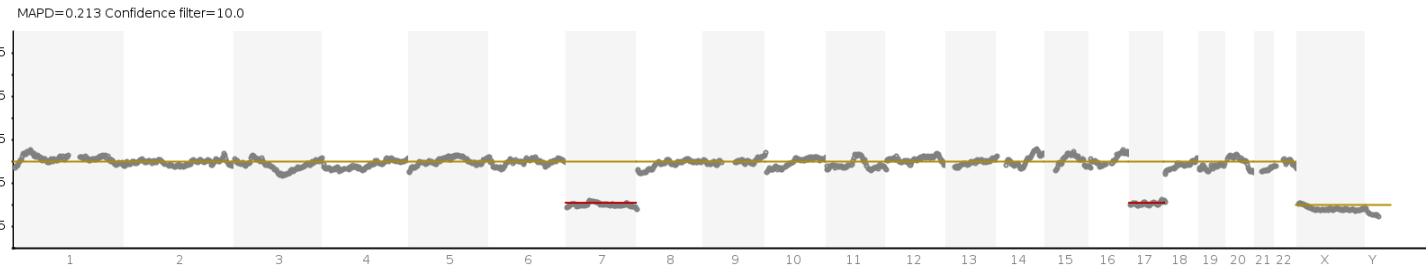

8-5

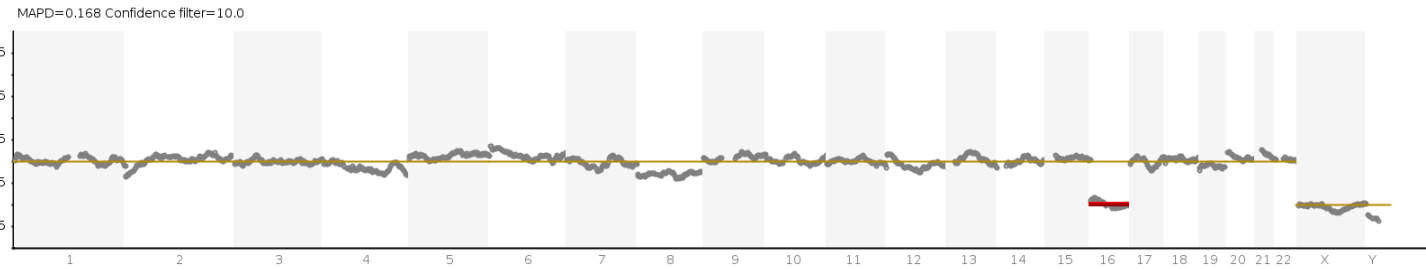

16-1

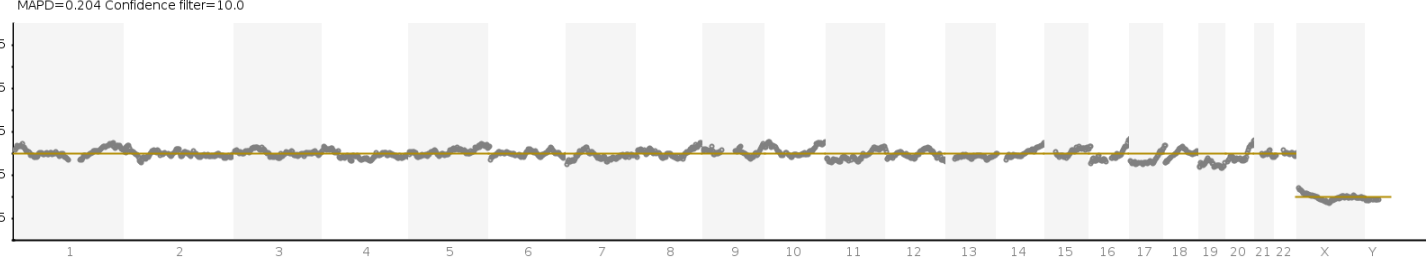

16-2

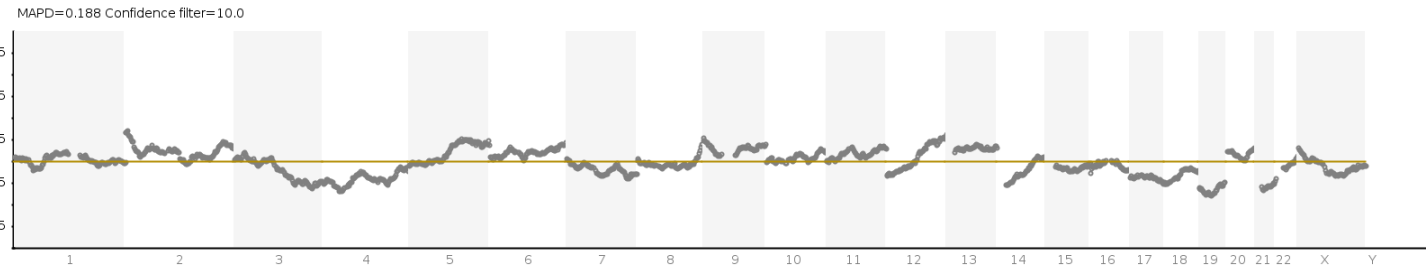

16-3

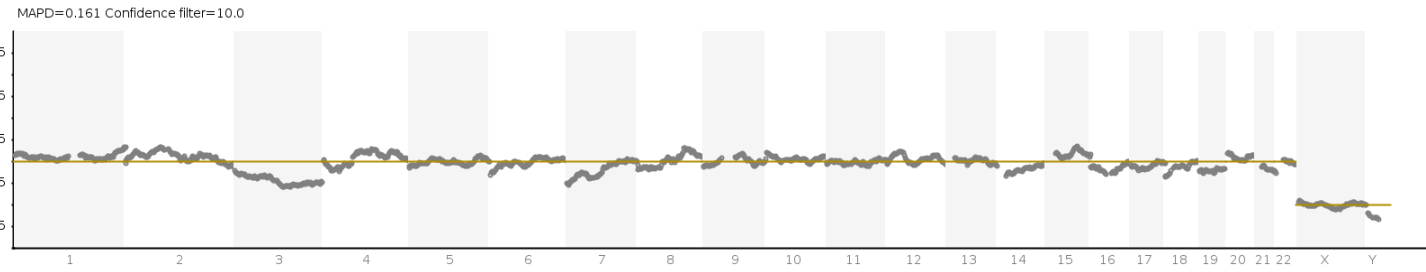

16-4

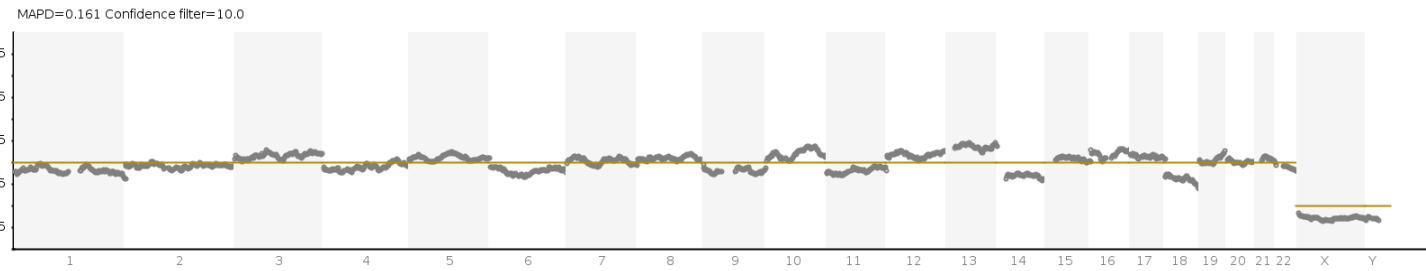

16-5

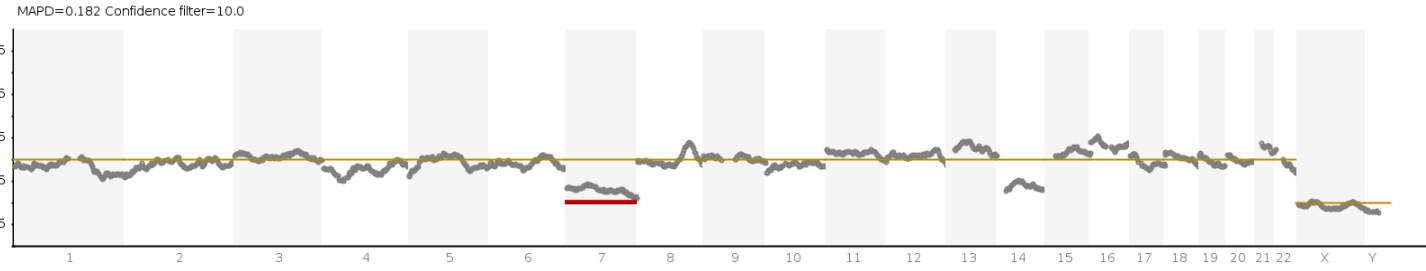

24-1

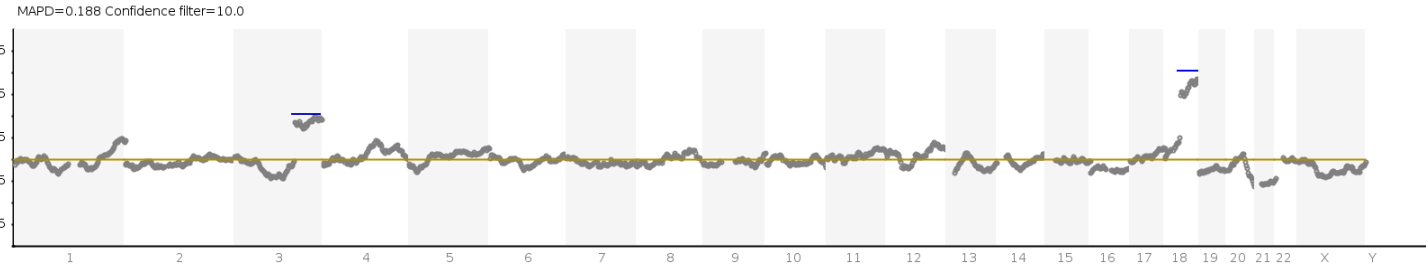

24-2

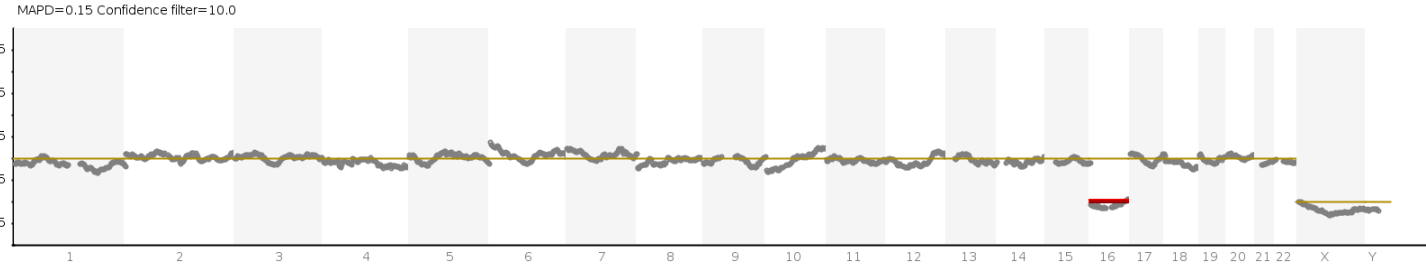

24-3

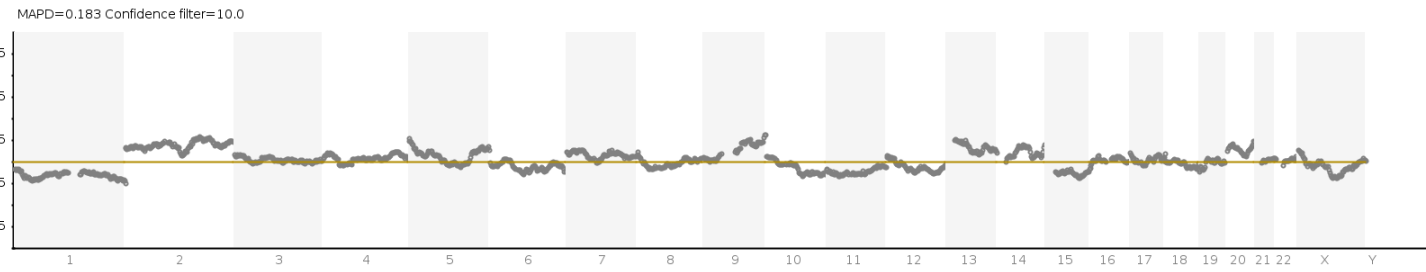

24-4

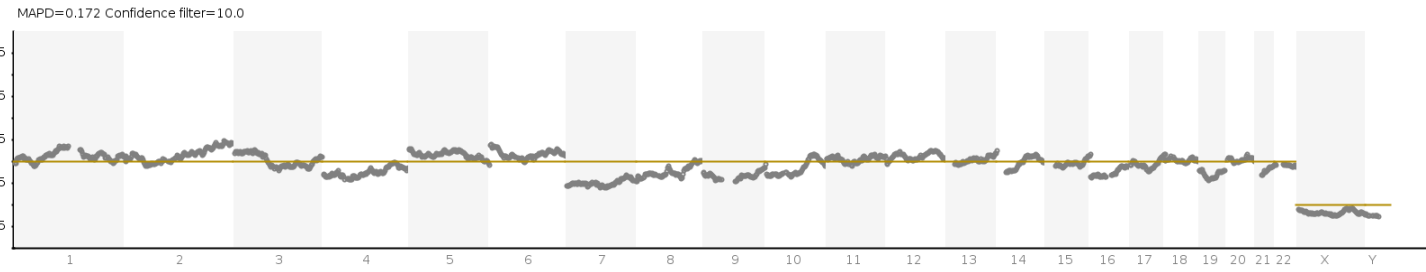

24-5

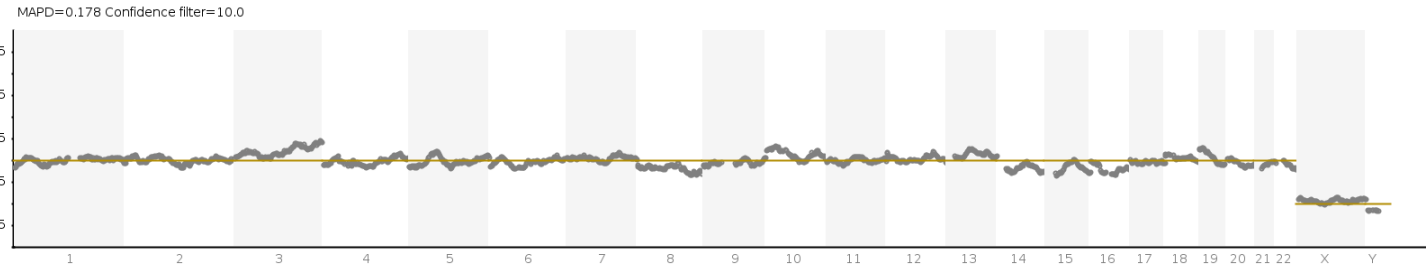

ZF-1

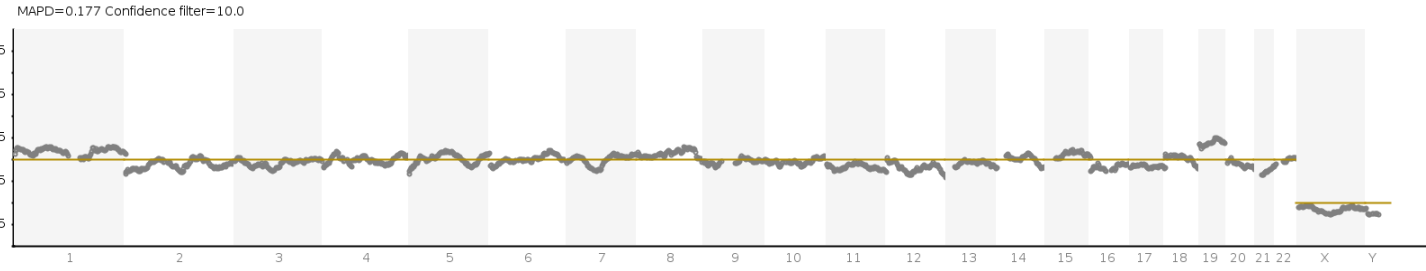

ZF-2

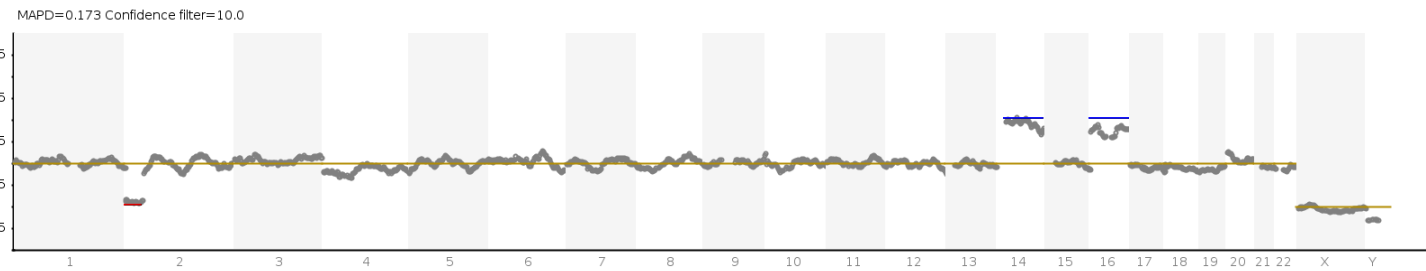

ZF-3

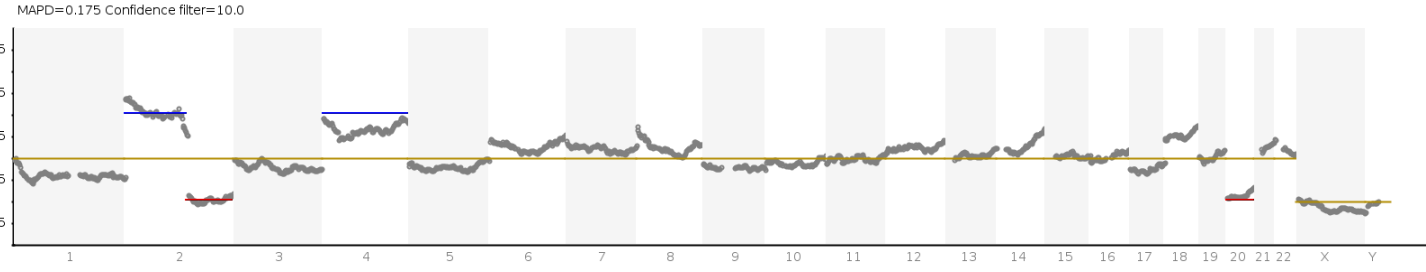

ZF-4

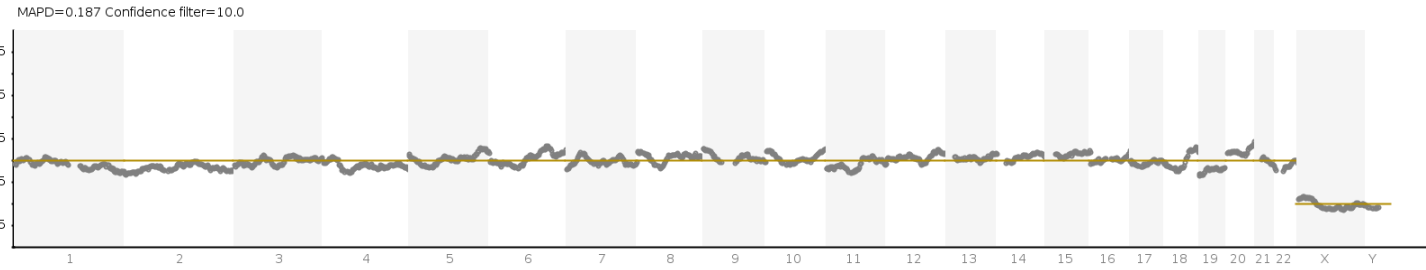

ZF-5

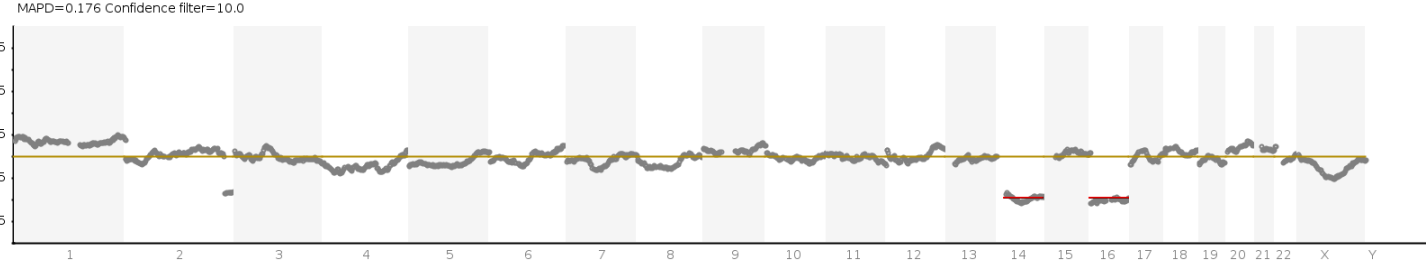

ZF-6

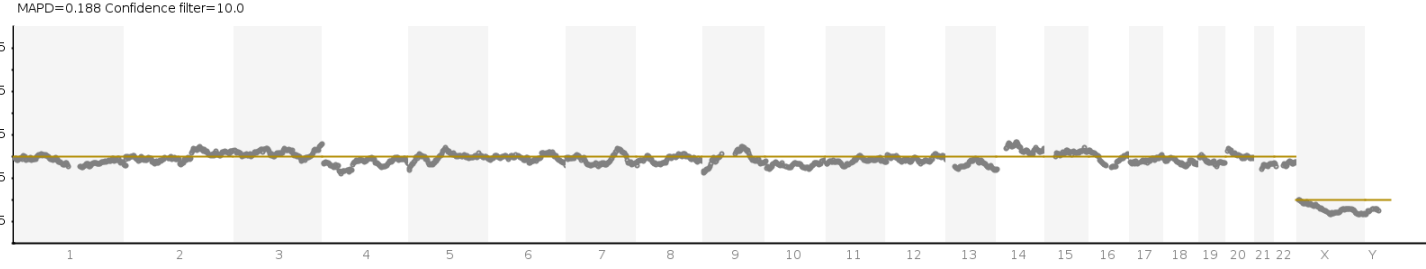

ZF-7

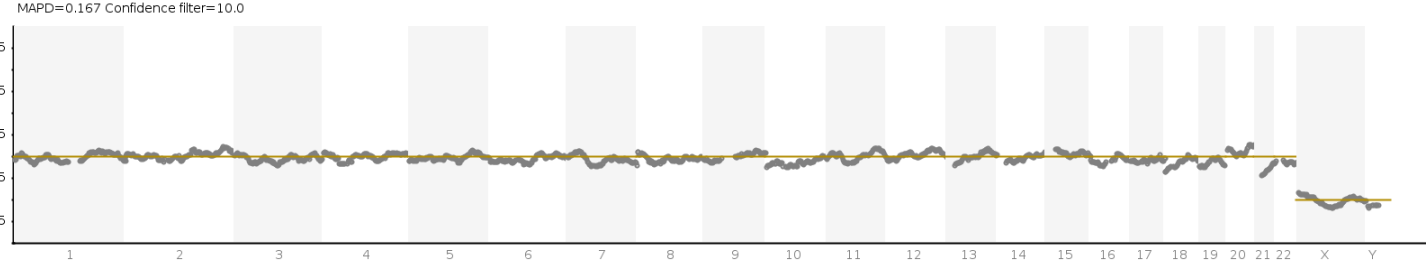

ZF-8

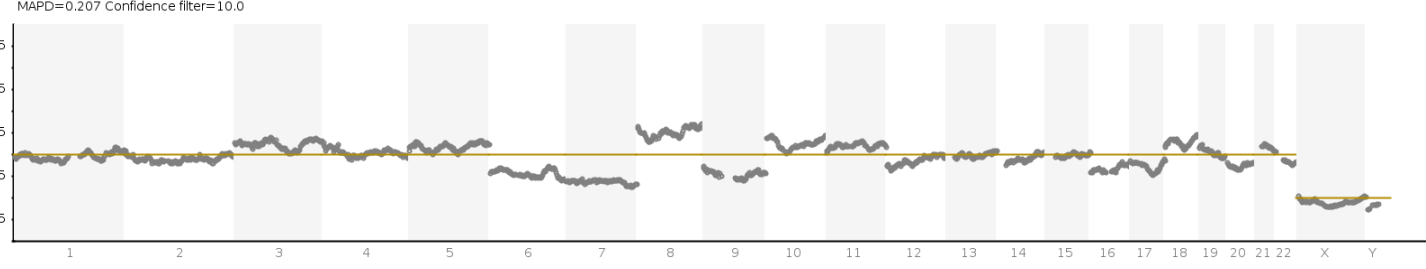

ZF-9

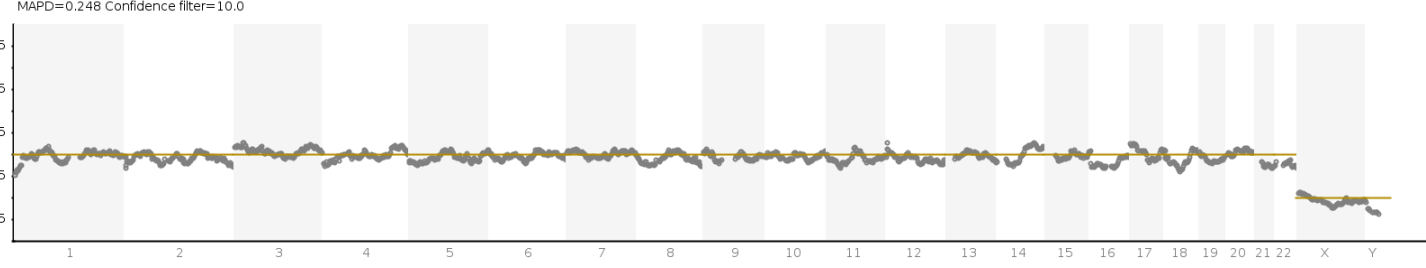

ZF-10

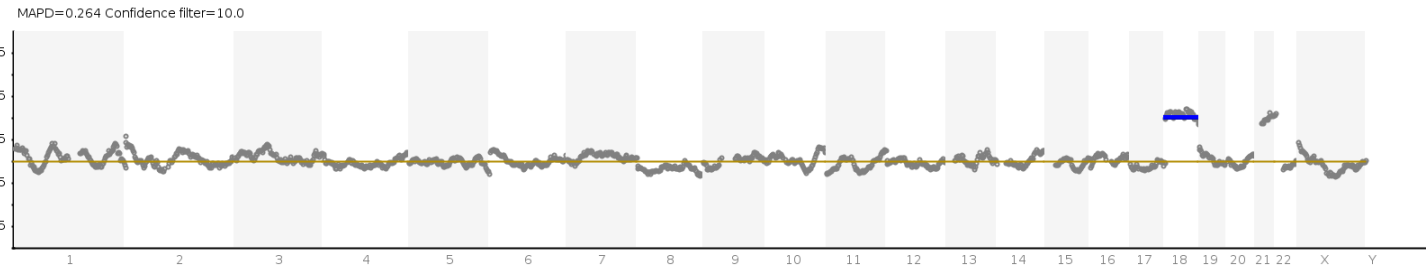

BL2-1

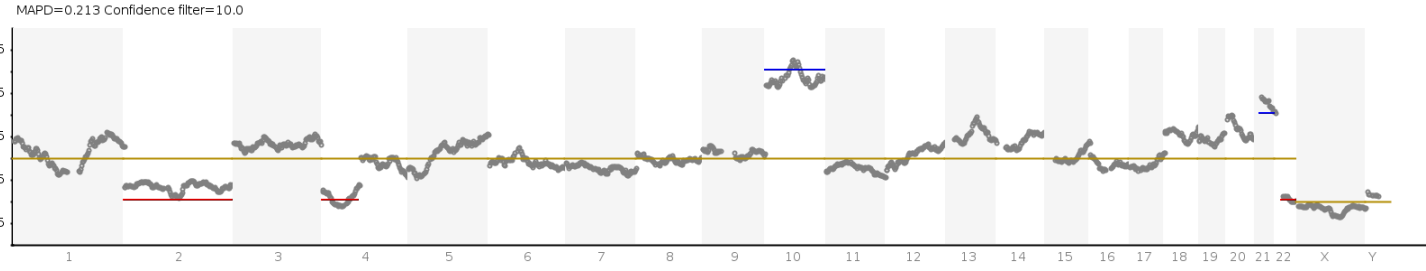

BL2-2

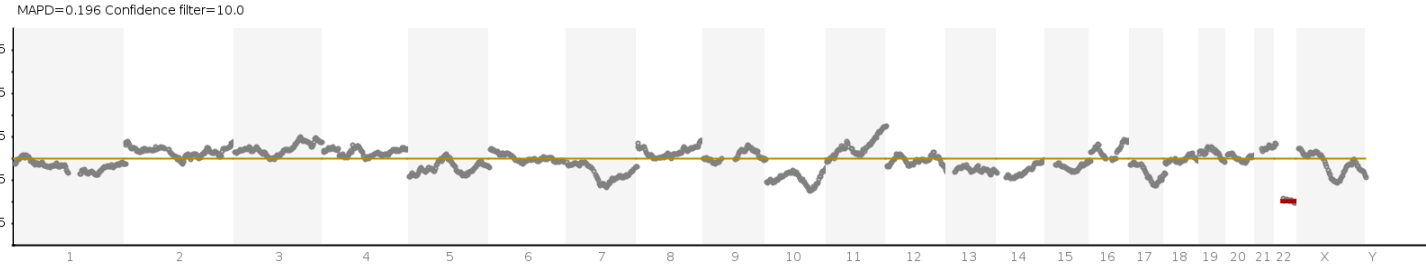

BL2-3

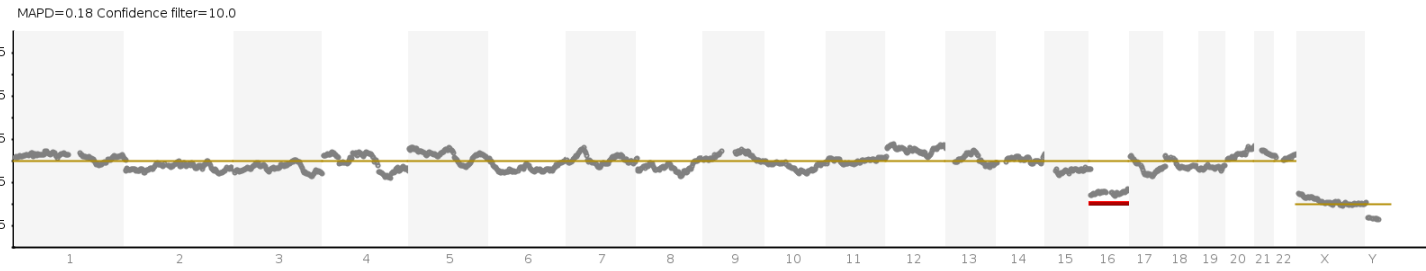

BL2-4

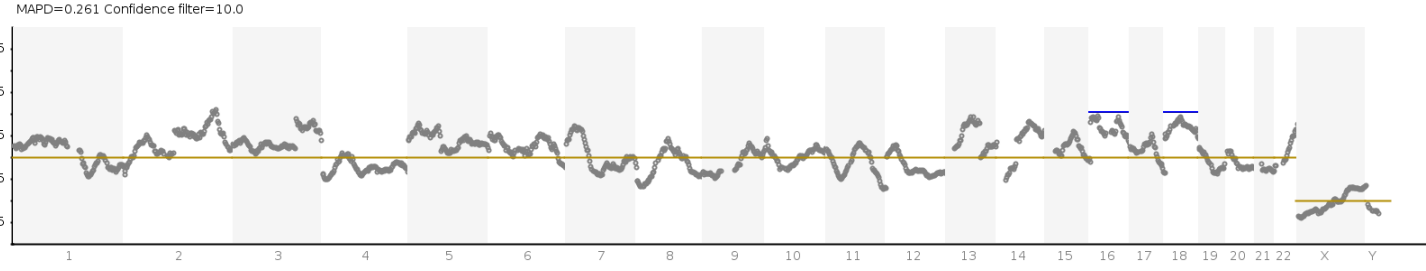

BL2-5

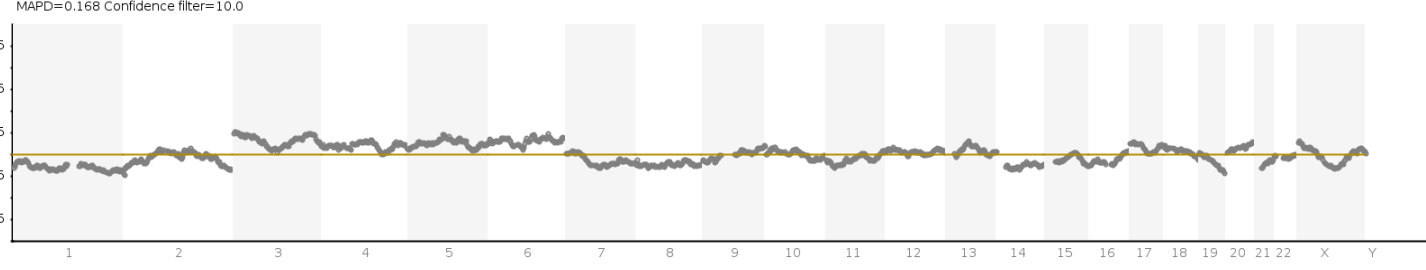

BL2-6

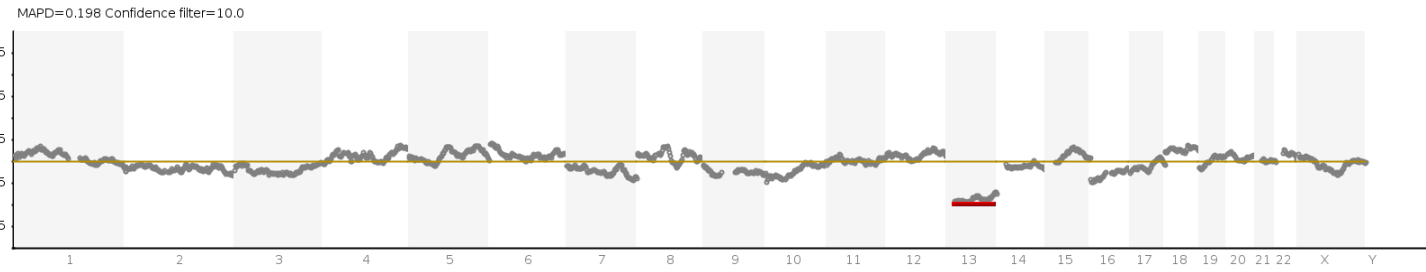

BL2-7

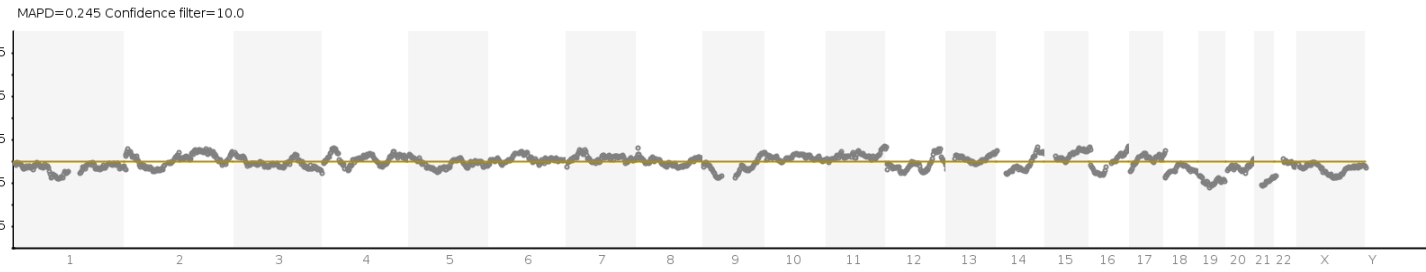

BL2-8

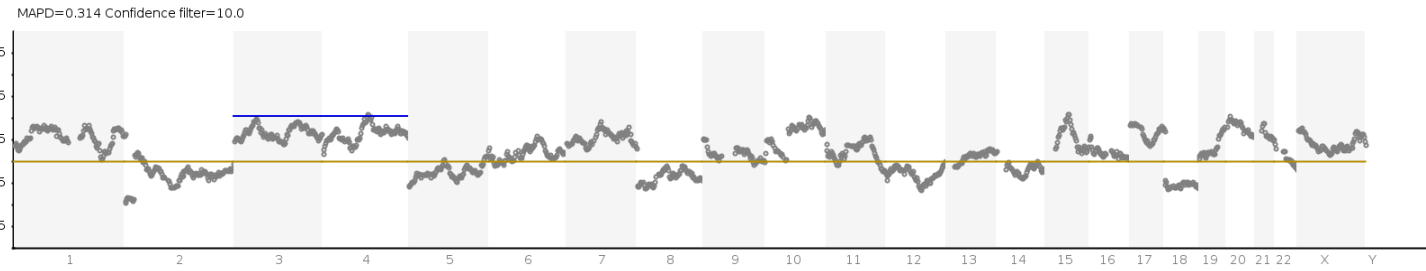

BL2-9

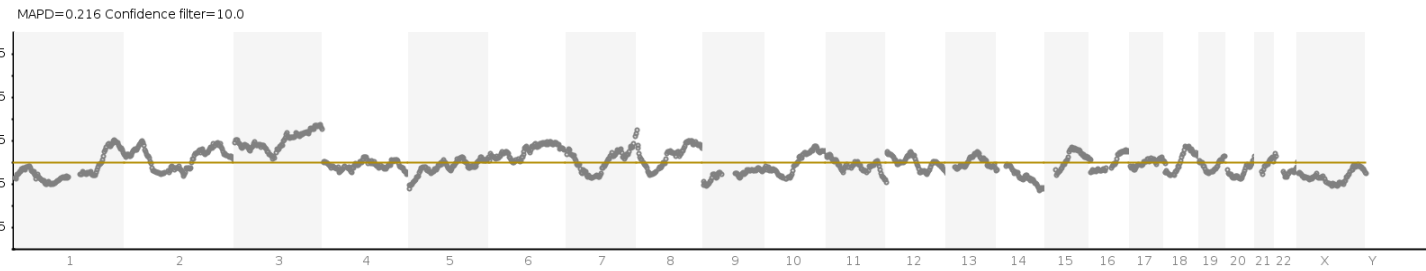

BL2-10

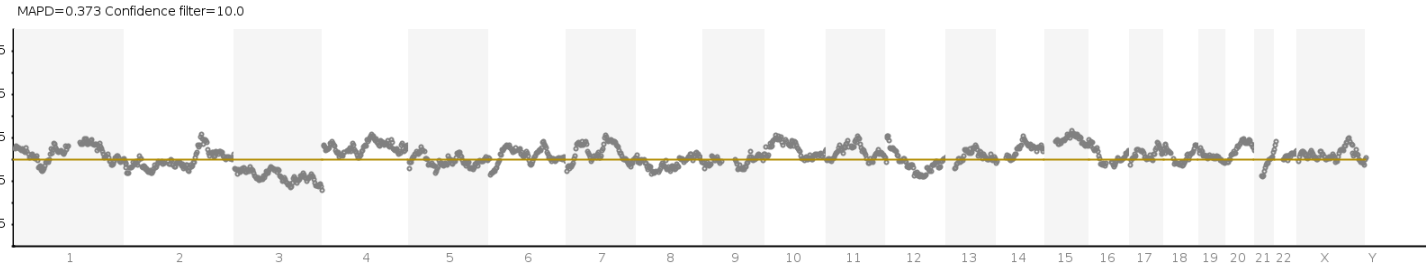

Supplement: Supplementary file 1 — Appendix S1: [file RMB2-23-e12604-s001.zip › rmb212604-sup-0002-FigureS2.pdf]

8-1

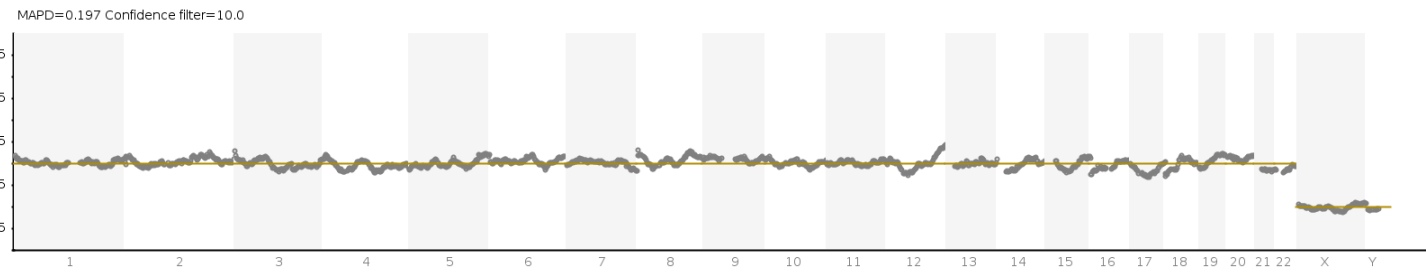

8-2

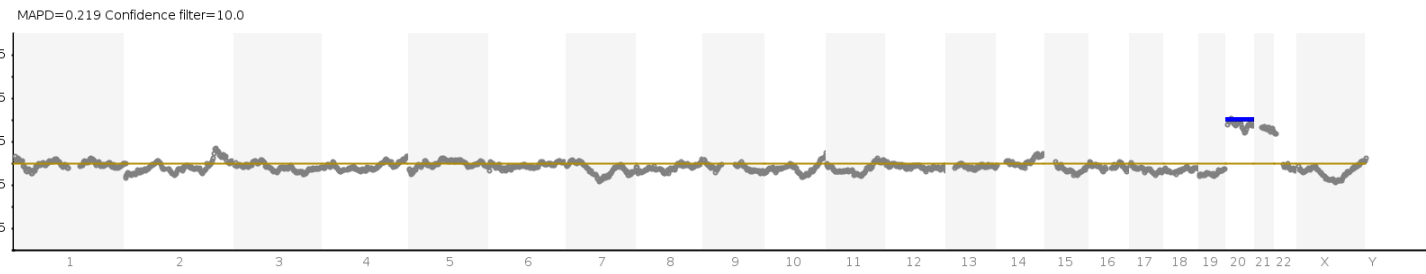

8-3

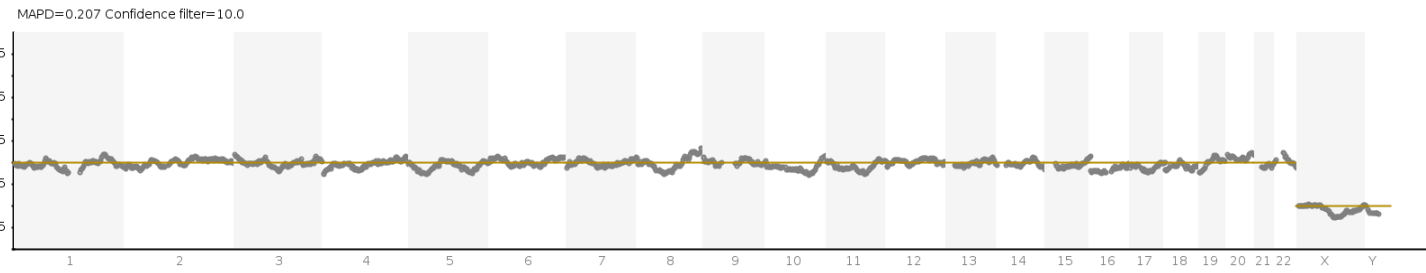

8-4

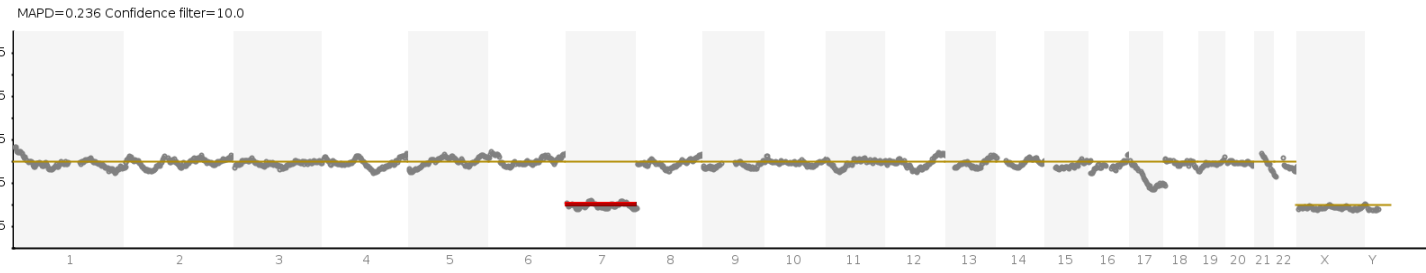

8-5

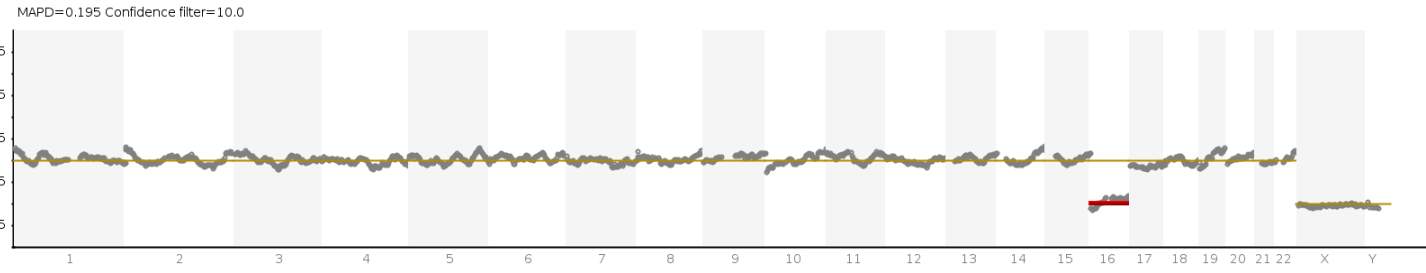

16-1

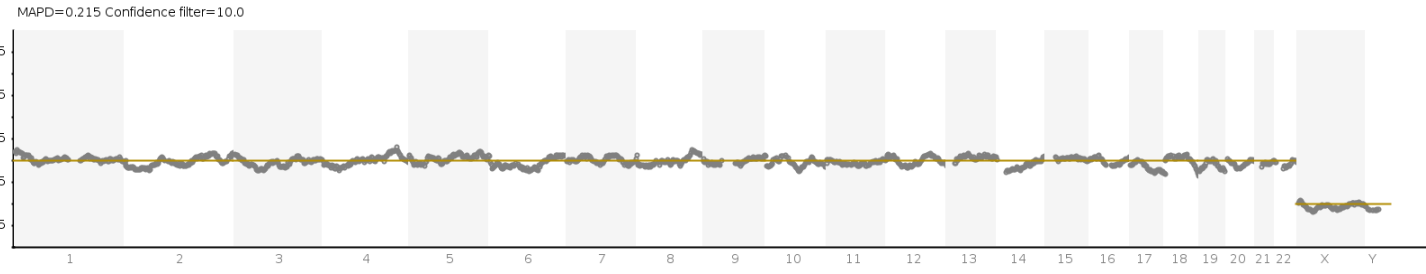

16-2

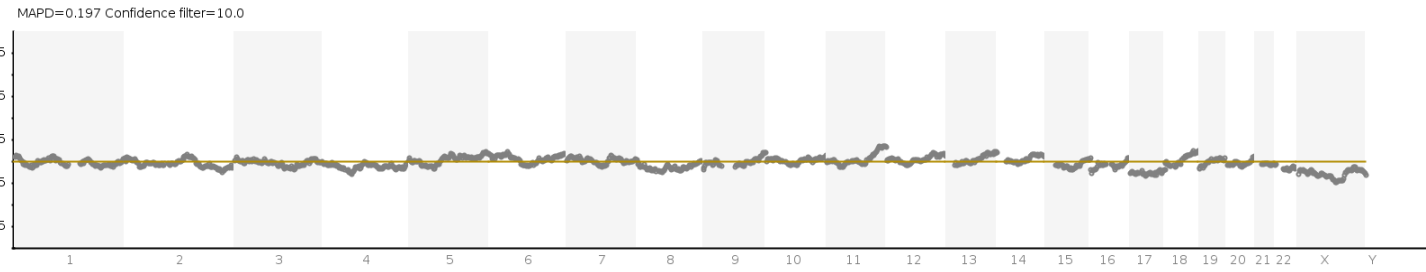

16-3

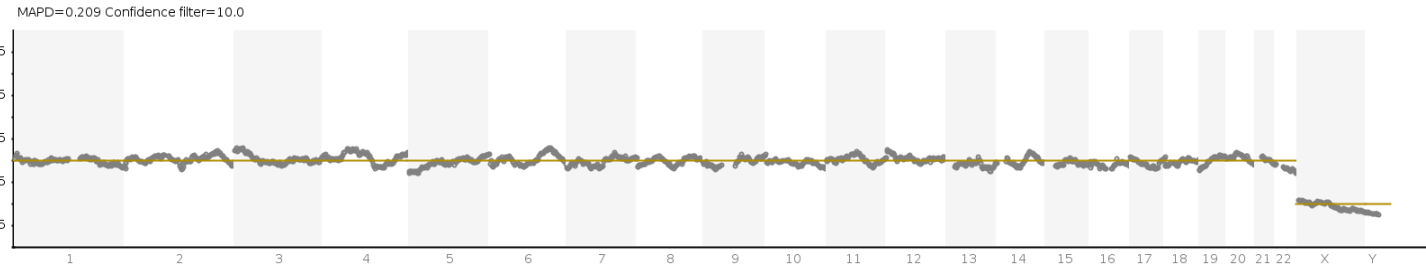

16-4

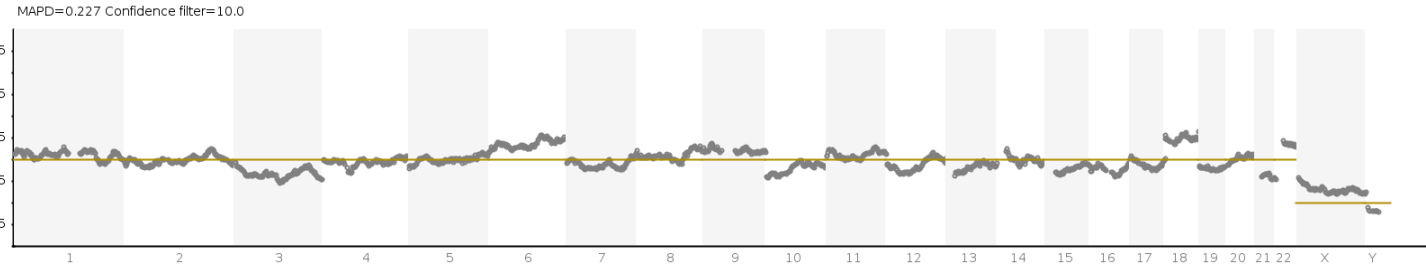

16-5

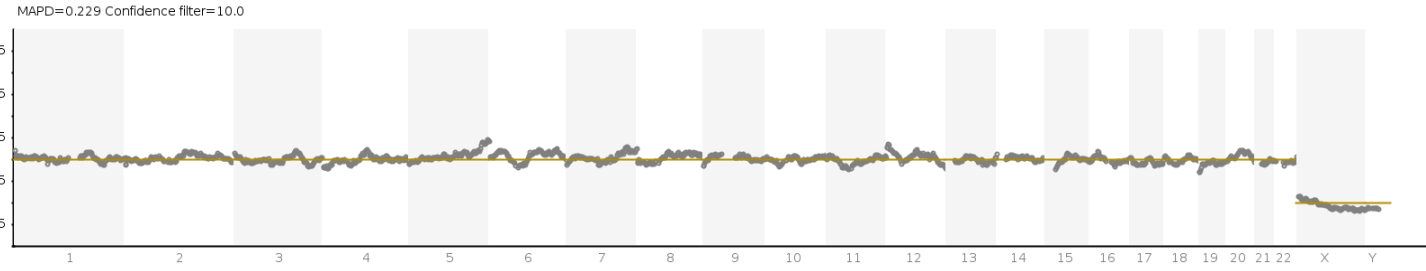

24-1

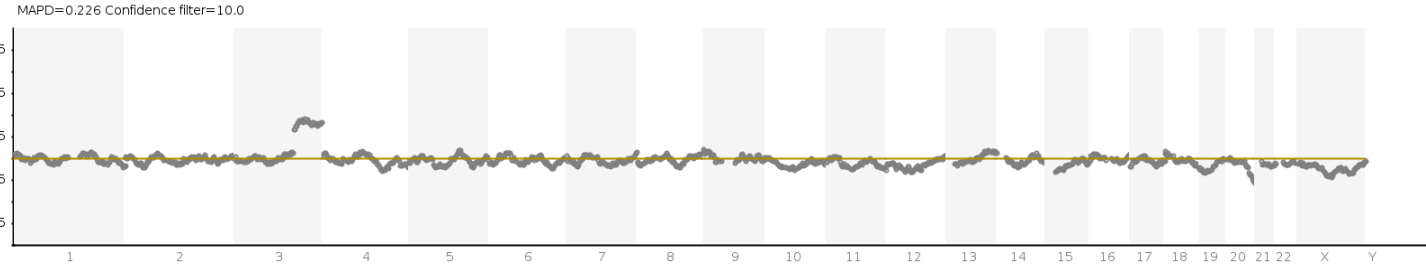

24-2

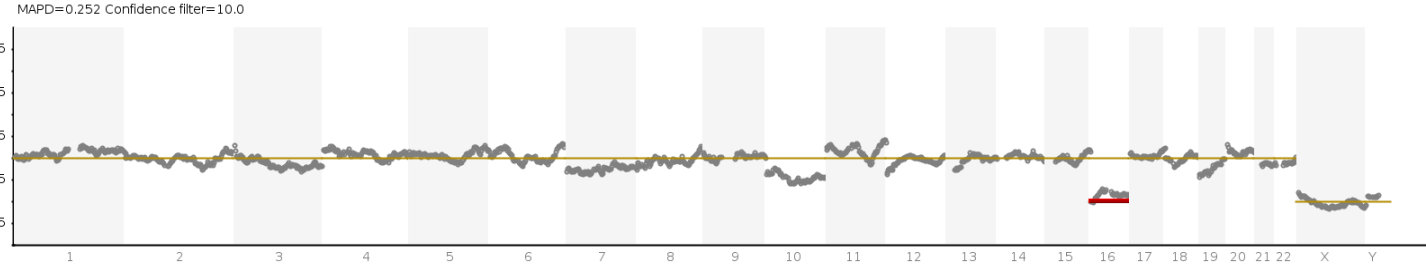

24-3

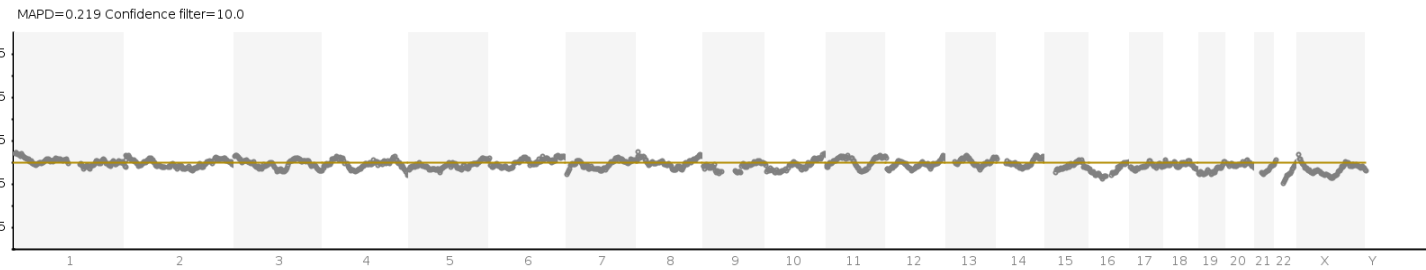

24-4

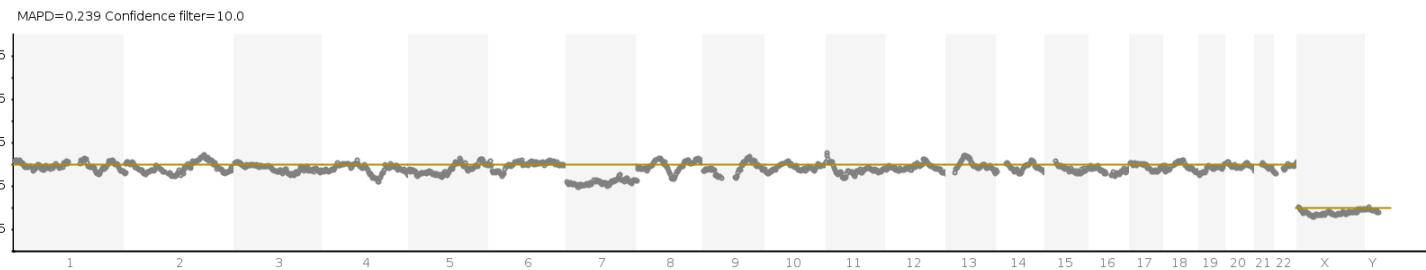

24-5

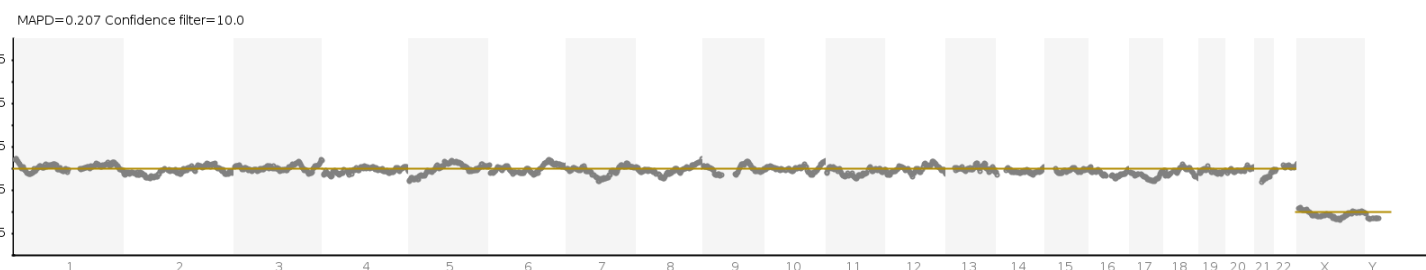

ZF-1

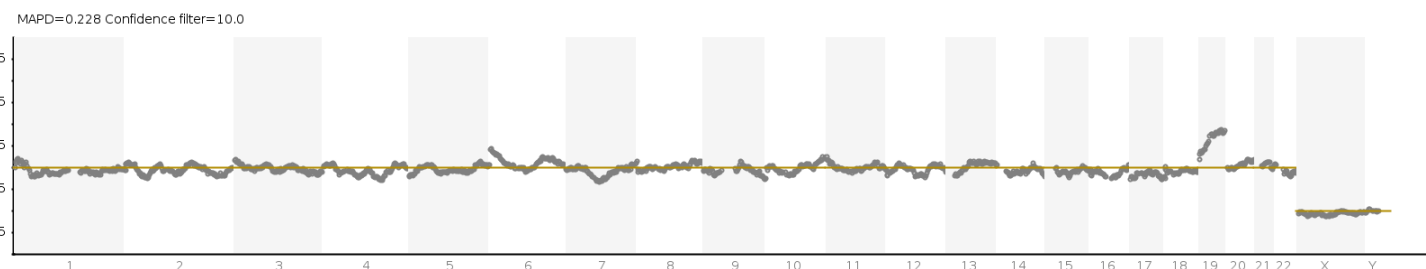

ZF-2

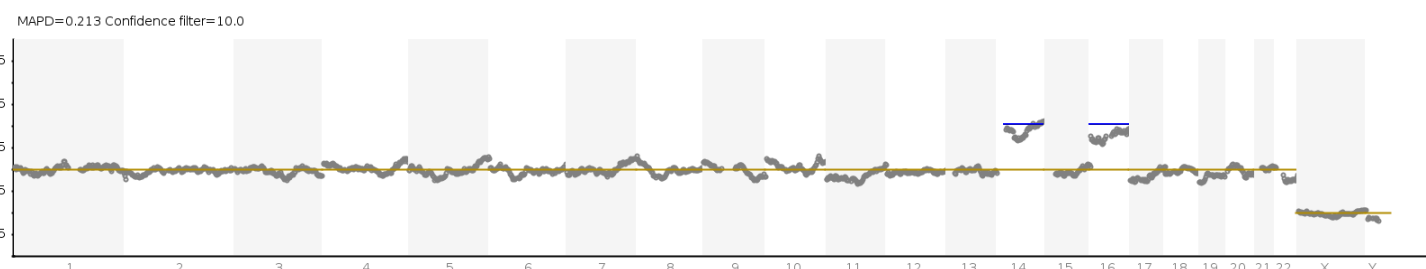

ZF-3

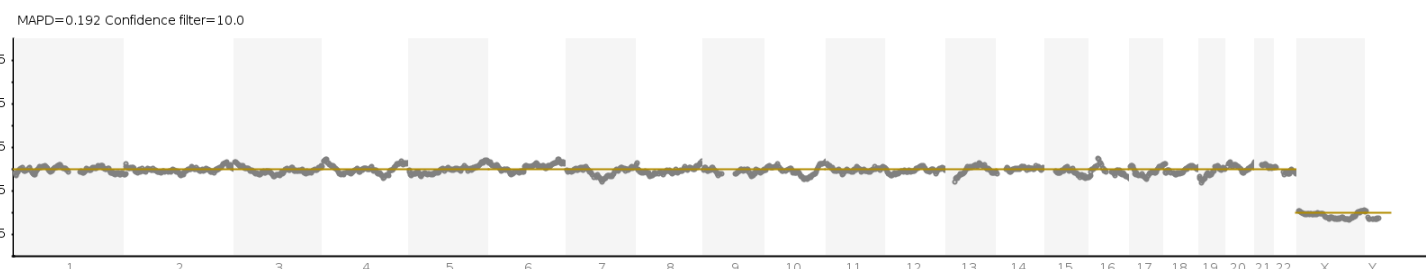

ZF-4

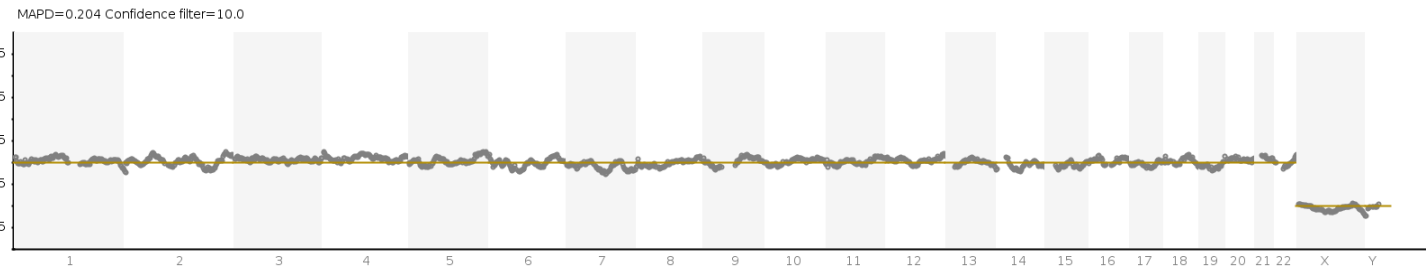

ZF-5

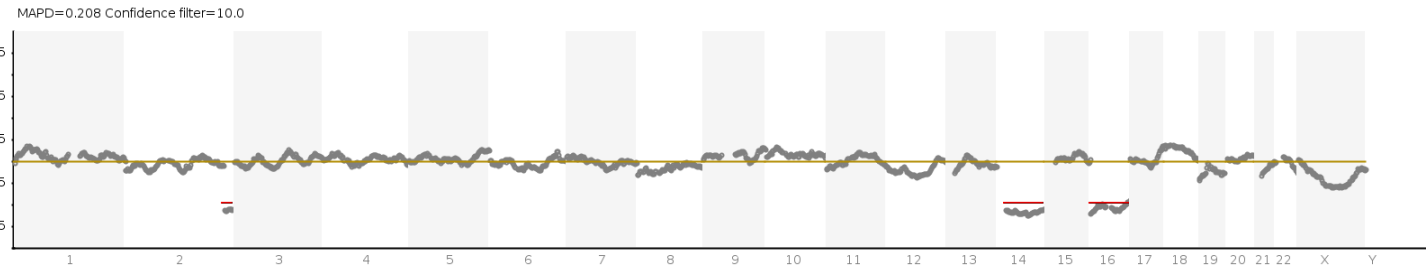

ZF-6

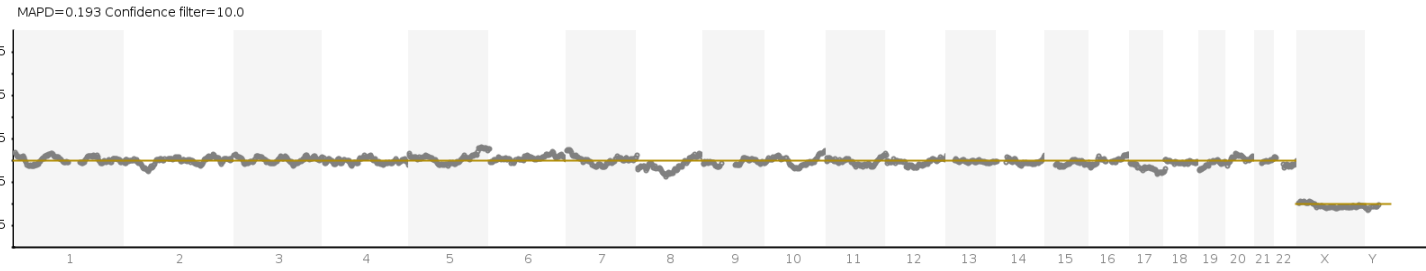

ZF-7

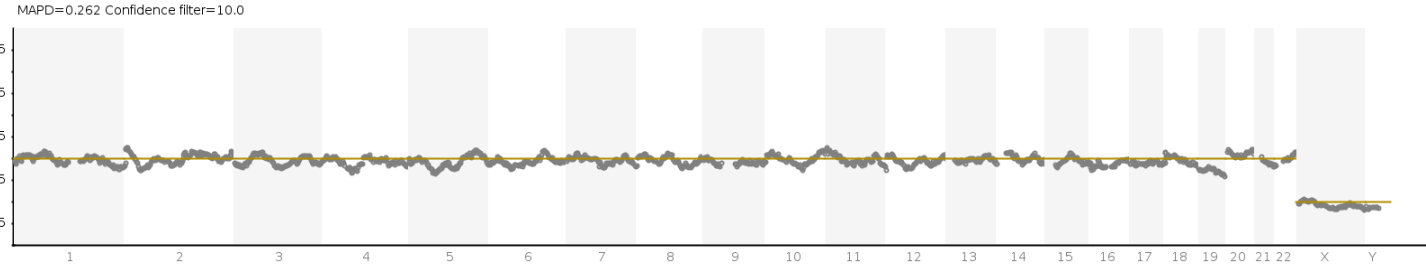

ZF-8

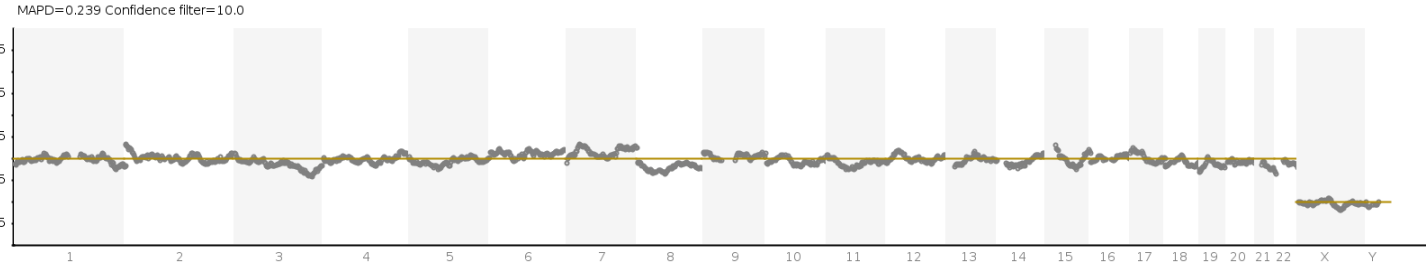

ZF-9

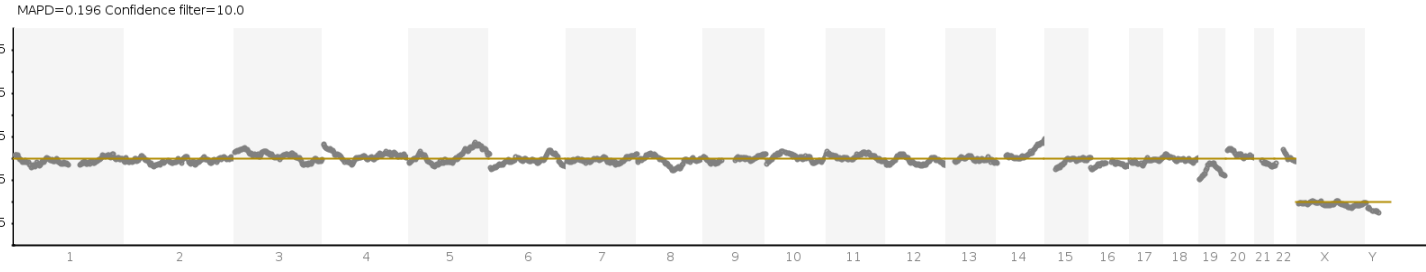

ZF-10

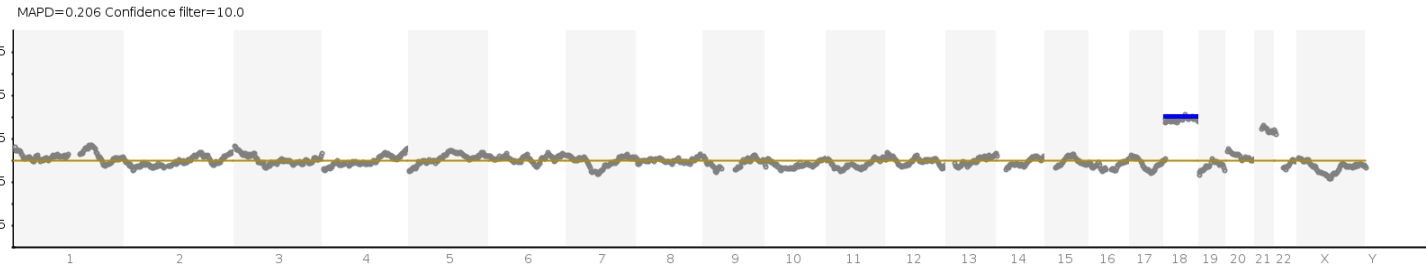

BL2-1

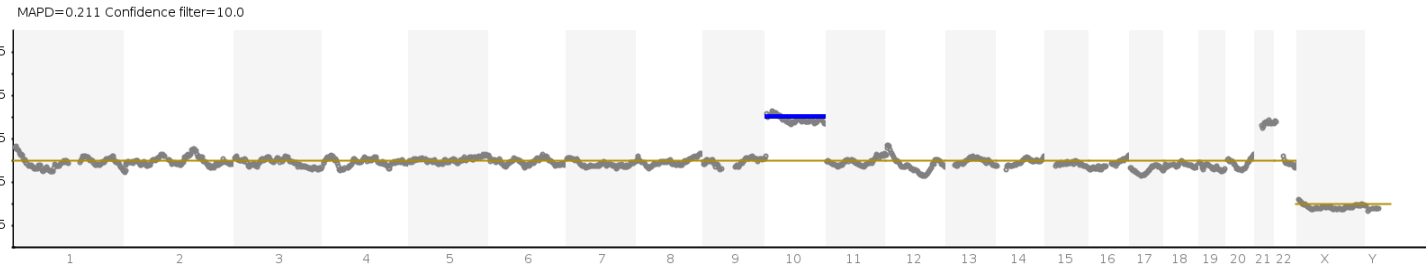

BL2-2

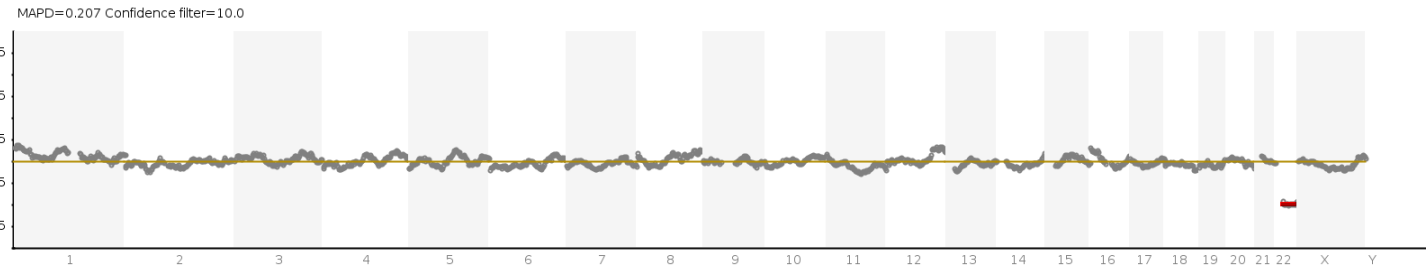

BL2-3

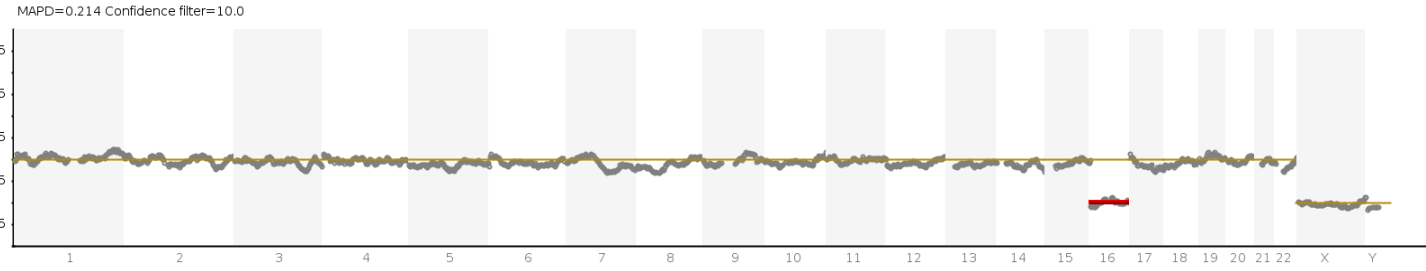

BL2-4

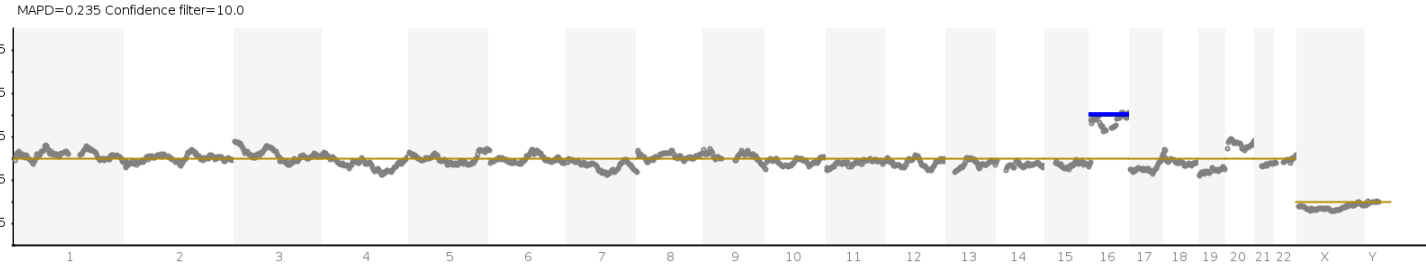

BL2-5

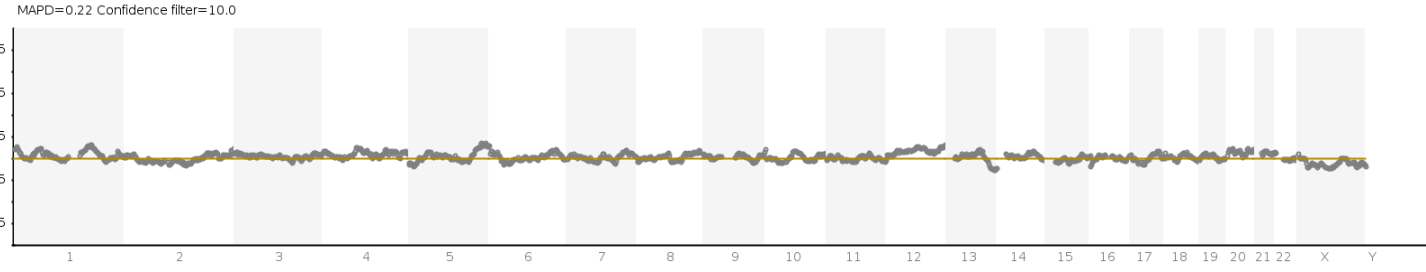

BL2-6

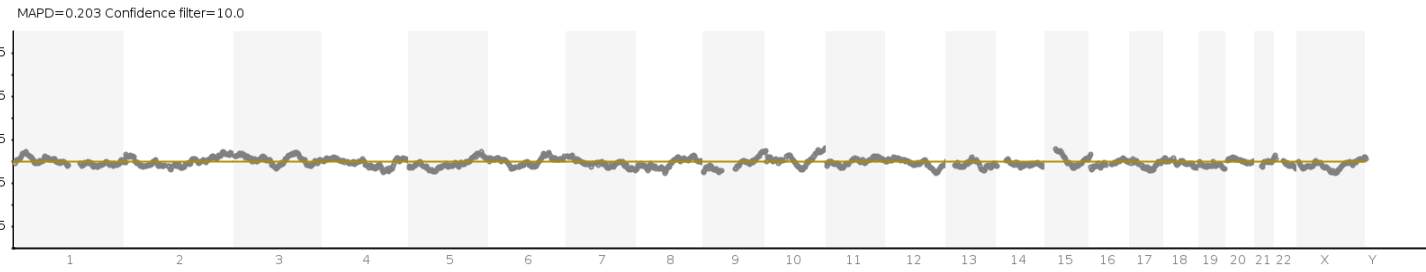

BL2-7

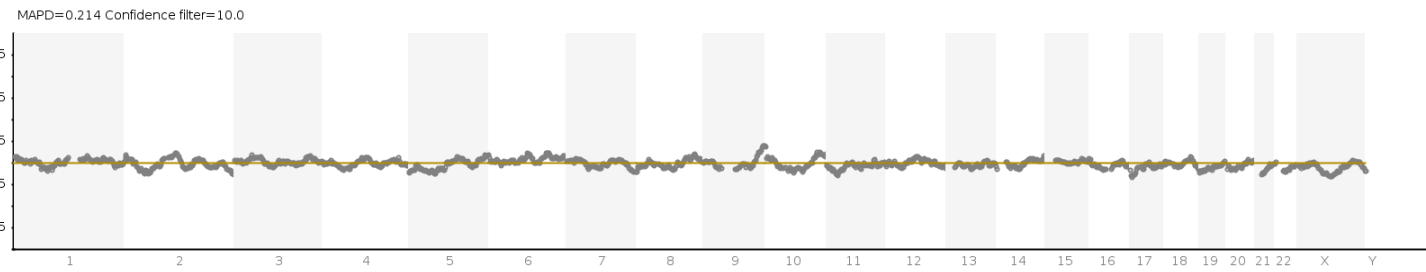

BL2-8

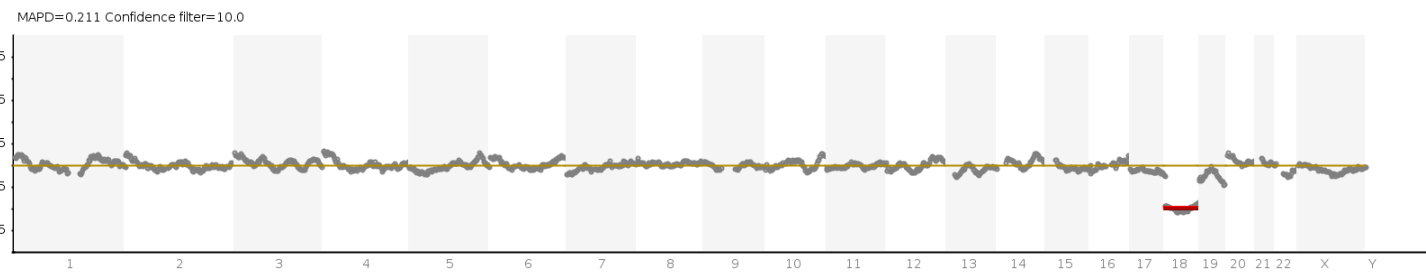

BL2-9

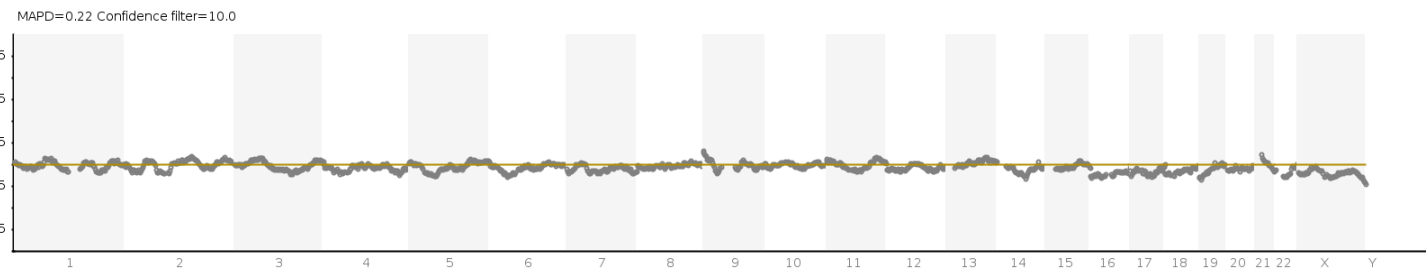

BL2-10

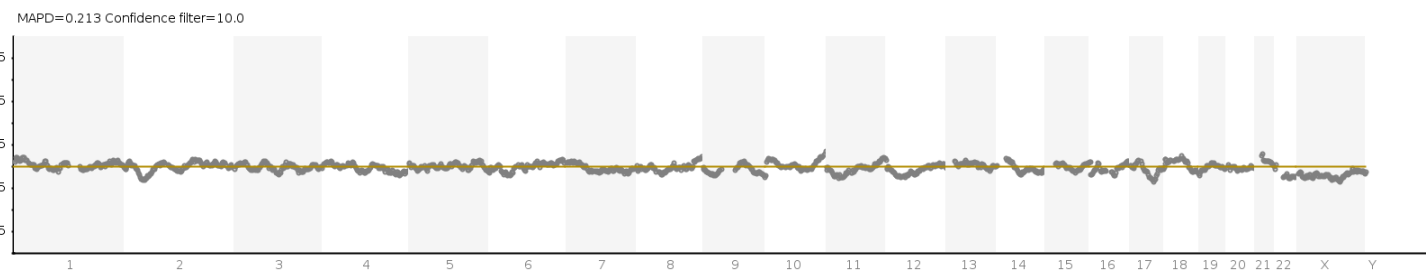

Supplement: Supplementary file 1 — Appendix S1: [file RMB2-23-e12604-s001.zip › rmb212604-sup-0003-FigureS3.pdf]
